# Characterization of *ddx4* and *dnd* Homologs in Snakeskin Gourami (*Trichopodus pectoralis*) and Their Expression Levels during Larval Development and in Gonads of Males and Females

**DOI:** 10.3390/ani12233415

**Published:** 2022-12-04

**Authors:** Chatsirin Nakharuthai, Somkiat Sreebun, Apinat Kabpha, Tran Vinh Phuong, Surintorn Boonanuntanasarn

**Affiliations:** 1 School of Animal Technology and Innovation, Institute of Agricultural Technology, Suranaree University of Technology, 111 University Avenue, Muang, Nakhon Ratchasima 30000, Thailand; 2 Department of Science Technology and International Relations, Hue University, 1 Dien Bien Phu St., Hue 49000, Vietnam

**Keywords:** snakeskin gourami, *Trichopodus pectoralis*, *ddx4*, *dnd*

## Abstract

**Simple Summary:**

Gene markers that are specific to germ cells are needed for the development of reproductive biotechnologies for fish. To provide valuable molecular information on *ddx4* and *dnd*, which can be applied for further monosex production in snakeskin gourami (*Trichopodus pectoralis*), this study cloned and characterized *ddx4* (*ddx4*) and *dnd* (*dnd1*) homologs and described their temporal expressions in snakeskin gourami (*Trichopodus pectoralis*). The expressions of *ddx4* and *dnd1* mRNAs were detectable only in the gonads, particularly in germ cells. The expression of *ddx4* was high during early larval development and decreased with increasing developmental age, whereas the expression of *dnd1* increased with developmental age. In adult fish, the expression levels of *ddx4* and *dnd1* were higher in the ovary than in the testis.

**Abstract:**

The purpose of this study was to clone and characterize *ddx4* and *dnd1* homologs in snakeskin gourami (*Trichopodus pectoralis*) and to determine their expression levels during larval development and in the gonads of males and females. Both cDNAs contained predicted regions that shared consensus motifs with the *ddx4* family in teleosts and the *dnd* family in vertebrates. Phylogenetic tree construction analysis confirmed that these two genes were clustered in the families of teleosts. Both *ddx4* and *dnd1* mRNAs were detectable only in the gonads, particularly in germ cells. These two genes were expressed during early larval development. The expression of *ddx4* was high during early larval development and decreased with increasing developmental age, whereas *dnd1* expression increased with developmental age. In adult fish, the expression levels of both genes were higher in the ovary than in the testis. Overall, these findings provide valuable molecular information on *ddx4* and *dnd,* and can be applied in future reproductive biological studies relating to sex dimorphism in snakeskin gourami.

## 1. Introduction

Snakeskin gourami (*Trichopodus pectoralis*, Regan, 1910) has become an economically important species for both the aquaculture and ornamental fish trades. Indeed, it is a species that is recommended for cultivation in planted freshwater tanks to create natural-environment aquaria. It is naturally distributed in Southeast Asia and is one of the most valuable commercial freshwater fish culture species in Thailand. The full life cycle of snakeskin gourami is completed in approximately 8–12 months. It reaches harvestable size during maturity, when males and females exhibit strong sexual dimorphism; the females grow larger than the males. To increase the productivity of snakeskin gourami, monosex cultures, such as all-female production, can be applied as a production tool. Although snakeskin gourami has been widely farmed, all-female production at the farm scale has not been achieved. Generally, monosex fish populations can be obtained using direct and/or indirect methods [1,2]. In the direct method, all-female fish can be obtained by feeding the fish a diet supplemented with β-estradiol (E2) at 200–300 mg/kg of food, which can produce feminized effects throughout the adult phase. To develop sustainable and reliable all-female production of snakeskin gourami, more biotechnological techniques to identify sex differences are required. DNA markers that are specifically expressed in gonads and differentially expressed in the testes and ovaries are required to distinguish and confirm genotypic sex, particularly during the age at which the phenotypic sex is not yet exhibited. For instance, sex-specific DNA markers were revealed and provided an approach for sex control in aquaculture in a previous study (reviewed in [3]). In particular, a male-specific DNA marker was applied to screen *cyp17a1-*deficient neomale carp (*Cyprinus carpio* L.) [4].

Several germ cell markers have been investigated and used in reproductive biological and biotechnological studies on vertebrates, including DEAD (Asp-Glu-Ala-Asp) box polypeptide 4 (*ddx4*; previously known as *vasa*), microRNA-mediated repression inhibitor 1 (*dnd1;* previously known as *dnd*), nanos protein (*nanos3*; previously known as *nanos*), piwi-like RNA-mediated gene silencing 1 (*piwil1*; previously known as *piwi*), sry-box transcription factor sox-3 (*sox3*), and deleted in azoospermia-like (*dazl*) [5,6,7,8,9,10,11,12]. Specifically, *ddx4* and *dnd* have been extensively used as DNA markers for germline and gonad development, sex determination, and sex differentiation in fish [9,13,14,15,16,17,18,19,20,21]. It has been reported that *ddx4* is responsible for a wide variety of functions, such as RNA binding, RNA splicing, RNA editing, ATP binding, and hydrolysis [22,23,24,25]. It is a maternally expressed gene and plays a pivotal role in the regulation of gonadal development [26]. Furthermore, *dnd1* is a maternal germline-specific gene that encodes an RNA-binding protein. It plays a vital role in the migration, survival, and proliferation of primordial germ cells (PGCs) during early embryonic development [15,27]. It prevents germ-cell-specific RNA degradation via the inhibition of miRNA function by binding to the 3′-untranslated regions (3′UTRs) of germ-cell-specific RNAs [28]. Both *ddx4* and *dnd* have been identified and their functions related to gonadal development and/or gametogenesis in several fish, including medaka (*Oryzias latipes*) [29], turbot (*Scophthalmus maximus*) [19], olive flounder (*Paralichthys olivaceus*) [9], gibel carp (*Carassius gibelio*) [13], starry flounder (*Platichthys stellatus*) [21], tilapia (*Oreochromis niloticus*) [30], brown-marbled grouper (*Epinephelus fuscoguttatus*) [31], striped catfish (*Pangasianodon hypophthalmus*) [7], and black rockfish (*Sebastes schlegelii)* [8].

Based on their specific expressions in the germline, the expressions of *ddx4* and *dnd* have been applied in several reproductive investigations into vertebrates. For example, the differential expression of *dnd* mRNA levels between males and females is applicable for sex identification in Xenopus [32], mice [33], medaka [29], and turbot [19]. In addition, in the hermaphrodite orange-spotted grouper, the upregulation of *dnd* during sex reversal suggests that *dnd* may play an important role in regulating sex reversal and spermatogenesis [9]. Moreover, in germ cell transplantation technology, *ddx4* has been used as a germ cell marker to validate transplanted germ cells of donors in recipient fish [34], and *ddx4* expression has been related to external hormones [35]. In other words, the specific expressions of *dnd* and *ddx4* in the germline have been established for use as gene markers in reproductive biotechnology. This study aimed to clone and characterize the full-length cDNAs of *ddx4* and *dnd1* from snakeskin gourami and demonstrate their specific expressions in gonads. The expression levels of both genes at different larval developmental stages were evaluated. In addition, sex differences in the expressions of both genes were determined at the adult stage.

## 2. Materials and Methods

### 2.1. Experimental Fish and Fish Sampling

Male and female snakeskin gourami broodstocks (8–9 months old) were obtained from a commercial farm in Samutr Sakhon, Thailand. The fish were maintained throughout the study in an earthen pond with an aeration system and a natural light/dark cycle at the Suranaree University of Technology Farm (SUT Farm; Nakhon Ratchasima, Thailand). The fish were fed to satiety with a commercial diet (40% crude protein, 6% fat) twice daily at 10:00 and 16:00 h. The study protocol was approved by the Ethics Committee of the Suranaree University of Technology Animal Care and Use Committee (approval no. A-17/2562). Mature males (n = 10, 130–150 g body weight) and females (n = 20; 140–170 g body weight) were selected for mating to obtain fish larvae. 

### 2.2. Fish Sampling and Total RNA Extraction

For the cloning of *ddx4* and *dnd1* and analysis of their expression levels in various tissues, male (135 g) and female (160 g) fish were euthanized with 1% clove oil, and the testes and ovaries, respectively, were subsequently sampled for total RNA extraction. In addition, 50 g samples, including gills, heart, intestine, kidney, liver, muscle, skin, and stomach, were collected for total RNA extraction. Total RNA was extracted from the sampled tissues (approximately 100 mg) using TRIzol reagent (Invitrogen, Carlsbad, CA, USA) and digested with RNase-free DNase I (Promega, Madison, WI, USA) according to the manufacturer’s instructions. 

### 2.3. Cloning of the Full-Length ddx4 and dnd1 cDNAs

Full-length *ddx4* and *dnd1* cDNAs were cloned using the total RNA (1 µg) extracted from the testes and ovaries. The 3′ and 5′ first-strand cDNAs were synthesized from the total RNA using a SMART™ RACE cDNA amplification kit (Clontech, Palo Alto, CA, USA) according to the manufacturer’s instructions. The full-length cDNA of *ddx4* was cloned using a nested polymerase chain reaction (PCR). For the 3′ end, two primer sets for *ddx4* (primary; Tpe-ddx4F1, nested; Tpe-ddx4F2) were designed according to the highly conserved amino acid sequences of other teleost *ddx4* genes (Supplementary File S1; Table 1). For the 5′ end, two gene-specific primers for *ddx4* (primary; Tpe-ddx4R1, nested; Tpe-ddx4R2) were designed based on the DNA sequences of the 3′ end of *ddx4* (Table 1). For the full-length *dnd1* cDNA, the partial cDNA of *dnd1* was cloned using degenerate primers (Tpe-dndF1 and Tpe-dndR1) (Table 1), which were designed according to the conserved amino acid sequences of teleost *dnd* cDNAs (Supplementary File S1). Subsequently, two gene-specific primers, Tpe-dndF2 and Tpe-dndR2 (Table 1), for the 3′ and 5′ ends, respectively, were designed based on the partial cDNA sequences of *dnd1* cDNA. Reverse and forward primers for the 3′- and 5′-end amplifications were obtained using the SMART™ RACE kit. 

For PCR, 2.5 µL of cDNA (synthesis using 1 µg of total RNA), 1 µL of dNTP mix (2.5 mM each), 10 pmol of each primer, 2.5 mM MgCl_2_, 1.0X LA Taq^TM^ buffer, and 1.25 U LA Taq (Takara Shuzo, Shiga, Japan) were used in a final volume of 25 µL. PCR was carried out at 95 °C for 5 min, followed by 35 reaction cycles of 45 s at 95 °C, 45 s at 55 °C, and 90 s at 72 °C. The final elongation step was carried out at 72 °C for 5 min. The PCR products of the expected sizes were isolated and purified using the QIAquick Gel Extraction Kit (Qiagen, Crawley, UK). The PCR-amplified DNA fragments were cloned into the pGEM-T Easy plasmid (Promega). At least five sequenced clones from partial and both cDNA directions were submitted for sequencing (Macrogen Inc., Korea). The plasmid containing the 3′ RACE-DNA fragment of *ddx4* (p3-*ddx4*) and partial *dnd1* cDNA (p*dnd1*) was used for further analysis. Multiple sequence alignment was conducted using the CLUSTAL W (http://ebi.ac.uk/Tools/clustalw/index.html, accessed on 1 October 2022) [36] and the MatGAT (Matrix Global Alignment Tool) Version 2.02 (http://bitincka.com/ledion/matgat/, accessed on 1 October 2022). Phylogenetic trees of Ddx4 and Dnd1 were constructed with 1000 bootstrap replications with the Mega 11 program using the UPGMA method [37,38].

### 2.4. Tissue Expression Analysis of ddx4 and dnd1

The first-strand cDNA was synthesized from 1 µg of the total RNA extracted from the gills, heart, intestine, kidney, liver, muscle, skin, and stomach using the ImPromIITM Reverse Transcription System Kit (Promega). Two pairs of gene-specific primers were designed to determine the expression levels of *ddx4* (Tpe-ddx4F2 and Tpe-ddx4R3) and *dnd1* (Tpe-dndF1 and Tpe-dndR4). The beta actin gene (*actb*) served as an internal control, and a pair of primers (β-actinF and β-actinR; Table 1) was used. Reverse-transcriptase (RT)-PCR analysis was performed in a total volume of 10 µL consisting of 1 µL of cDNA template, 1 µL of dNTP mix (2.5 mM each), 10 pmol of each primer, 2.5 mM MgCl_2_, 5X GoTaq Flexi buffer, and 0.25 U GoTaq DNA polymerase (Promega). The RT-PCR analysis was performed with an initial denaturation at 95 °C for 5 min, followed by 35 reaction cycles of 45 s at 95 °C, 30 s at 55 °C, and 30 s at 72 °C. The final elongation step was carried out at 72 °C for 5 min. The plasmids p3-*ddx4*, p*dnd*, and pActin, which contain partial cDNA of *actb* [39], were used as positive controls to determine *ddx4*, *dnd1*, and *actb*. The PCR products of *ddx4*, *dnd1*, and *actb* were verified using agarose gel electrophoresis and RedSafe™ Nucleic Acid Staining (JH Science, iNtRON Biotechnology, WA, USA). 

### 2.5. Histological Study and In Situ Hybridization

In order to investigate the expression levels of *ddx4* and *dnd1* in testicular and ovarian cells, immature and mature fish were used. Immature males (90 g) and females (120 g) and mature males (130 g) and females (160 g) were euthanized using 1% clove oil. The testis and ovary samples were fixed in Bouin’s solution for 18 h at 4 °C. Subsequently, the solution was replaced with 75% ethanol and samples were stored at 4 °C until analysis. For histological analysis, the fixed tissues were embedded in paraffin wax and cut serially into 5 µm sections. The paraffin sections were dewaxed, dehydrated, and stained with hematoxylin and eosin (H&E) or subjected to in situ hybridization using the antisense or sense probes of *ddx4* and *dnd1*. 

To produce the antisense and sense RNA probes, the plasmids p3-*ddx4* and p*dnd* were linearized with *Apa*I and *Sal*I, respectively. In vitro transcription of antisense and sense RNA probes was performed using digoxigenin (DIG)-labeled uridine triphosphate (UTP) (Roche Diagnostics, Mannheim, Germany) with SP6 and T7 RNA polymerase (Promega), respectively, according to the manufacturer’s protocol. 

In situ hybridization was performed as described in [5], with some modifications. After RNA fixation, permeabilization, and acetylation steps, the sections were hybridized at 65 °C overnight in hybridization buffer containing 1.5 µg/mL antisense or sense probes, 50% formamide, 100 µL of 2x saline solution citrate buffer (0.3 M sodium chloride, 0.03 sodium citrate, pH 4.5), 50 µg/mL yeast tRNA, 50 µg/mL heparin, 1% sodium dodecyl sulfate, and 10% dextran sulfate. Subsequently, the sections were washed to remove the excess probes. The sections were incubated with the Fab fragment of an anti-DIG alkaline-phosphatase-conjugated antibody (Roche Diagnostics). 

For colorimetric detection, nitroblue tetrazolium/levamisole and 5-bromo-4-chloro-3-indolyl phosphate (Roche Diagnostics) were used to develop a blue color according to the manufacturer’s instructions. Subsequently, the sections were counterstained with Nuclear Fast Red (Vector Laboratories, Newark, CA, USA).

### 2.6. Fish Breeding, Larva Sampling, and Fish Sampling

Fish larvae were produced as described in [38]. Briefly, the breeding pond (replication, n = 5; 2.0 m × 2.0 m × 0.8 m), which contained cleaned floating aquatic plants for bubble nest building, was prepared with gentle aeration. Mature males and females (at a 1:2 male-to-female sex ratio) were selected and transferred to each breeding pond. For mating, the female fish were first injected with a mixture of luteinizing-hormone-releasing hormone (LHRH) analog (LHRHa) (Suprefact, Hoechst, Germany) at 15 µg/kg of body weight and domperidone (Motilium-M, OLIC, Bangkok, Thailand) at 10 mg/kg of body weight. Twelve hours later, the second hormone mixture containing LHRHa at 20 µg/kg of body weight and domperidone at 10 mg/kg of body weight was injected into the female fish. The male fish were injected with a hormone mixture containing LHRHa at 20 µg/kg of body weight and domperidone at 10 mg/kg of body weight. A bubble nest containing fertilized eggs was observed 14 h later. Hatching occurred 24 h later, and five pooled larval samples (50 mg/replication; n = 5) at the ages of 3, 5, 7, 9, 11, 13, 15, 21, 28, and 35 days post-hatching (dph) were collected by euthanizing them in ice-cold water and stored at −80 °C for RNA extraction. At 7 dph, the swim-up fry of each replicate was randomly selected and transferred into a nursing pond for continued growth until adulthood.

The fish larvae of each replication were cultured through the adult stage to investigate the effects of sex on the expressions of *ddx4* and *dnd1.* For the nursing facilities, five hapas (fine mesh aquatic dip net cages: 1 m × 1 m × 0.8 m) were placed in a cement pond (10 m × 5 m × 0.8 m) with aeration and a 12:12 light/dark cycle. The swim-up fry (500 fry/broodstock [replicate]; five replicates) were transferred and randomly distributed in each hapa. The fish were fed the experimental diet for three months. Subsequently, the experimental fish (20 experimental fish) were transferred to a hapa (2 m × 2 m × 1 m), which was located in an earthen pond (0.08 ha) with a 12:12 light/dark cycle. The fish were hand-fed a commercial diet (protein 35%, lipids 4%) twice daily to satiety for eight months (adult; at 8 months of age). For sampling, two female and two male fish in each replicate (female; n = 10, male; n = 10) were selected. The fish were euthanized with 1% clove oil, and, subsequently, either the testis or ovary was collected and stored at −80 °C for RNA extraction. The air and water temperatures were measured daily during the experimental period and recorded as 19.0–35 °C and 21.5–27.5 °C, respectively. Throughout the fish culture period, the dissolved oxygen (DO) and pH values were determined weekly using a DO meter (Pro20i-1P, YSI, OH, USA) and a pH meter (pH 1200, YSI, OH, USA), respectively, and were within the acceptable ranges of 3.78–6.80 mg L^−1^ and 6.80–7.84, respectively. 

### 2.7. Quantitative Expression Analysis of ddx4 and dnd1 mRNAs during Larval Development and in Adult Male and Female Fish

#### 2.7.1. Total RNA Extraction and cDNA Synthesis

The total RNA was extracted from 50 mg of the larvae, testes, or ovaries using TRIzol reagent (Invitrogen) and subsequently treated with RNase-free DNase I (Promega) according to the manufacturer’s instructions. Subsequently, first-strand cDNA was synthesized from 1 µg of the total RNA using an ImProm-II^TM^ Reverse Transcription System kit (Promega). 

#### 2.7.2. Quantitative RT-PCR Analysis

To quantitatively analyze the *ddx4* and *dnd1* mRNAs during fry development and at the adult stage, real-time quantitative RT-PCR (qRT-PCR) amplification (in triplicate) was carried out using a LightCycler^®^ 480 SYBR Green I Master Mix (Roche Applied Science, Indianapolis, IN, USA). For normalization, *actb* was used as the internal reference. The primers and annealing temperatures used for *ddx4*, *dnd1*, and *actb* are listed in Table 1, which generated amplicons of 158, 202, and 95 base pairs in length, respectively. The amplification efficiency (E) for *ddx4*, *dnd1*, and *actb* ranged between 99.39 and 106.15%. Each PCR was performed in a final volume of 10 µL consisting of 1 µL of cDNA template (synthesis using 1 µg of total RNA) or distilled water (negative control), 5 µL of LightCycler^®^ 480 SYBR Green I Master Mix, 1 μL of 5 µM of each primer, and 2 μL of distilled water. The PCR products were pre-incubated for 5 min at 95 °C, followed by 40 amplification cycles at 95 °C for 15 s, 55 °C for 15 s, and 72 °C for 15 s. The comparative cycle threshold (CT) method was used to analyze the data. Upon completion of amplification, PCR was performed to analyze the melting curve. External standard curves for *ddx4*, *dnd1*, and *actb* were generated using plasmids containing their respective cDNA fragments, as described in Section 2.7.2, with known copy numbers. Subsequently, the mRNA level of each gene was normalized to the expression level of *actb* using the following equation: log(copy number of *ddx4* or *dnd1*)/log (copy number of *actb*). Statistical analysis was performed using SPSS for Windows, version 25 (SPSS Inc., Chicago, IL, USA). The normalized *ddx4* and *dnd1* expressions were subjected to one-way analysis of variance (ANOVA), followed by Tukey’s procedure to rank the groups when significant differences (*p* < 0.05) were observed among the groups. 

## 3. Results

### 3.1. Molecular Cloning and Characterization of ddx4 and dnd1 in Snakeskin Gourami

**Figure 1:**
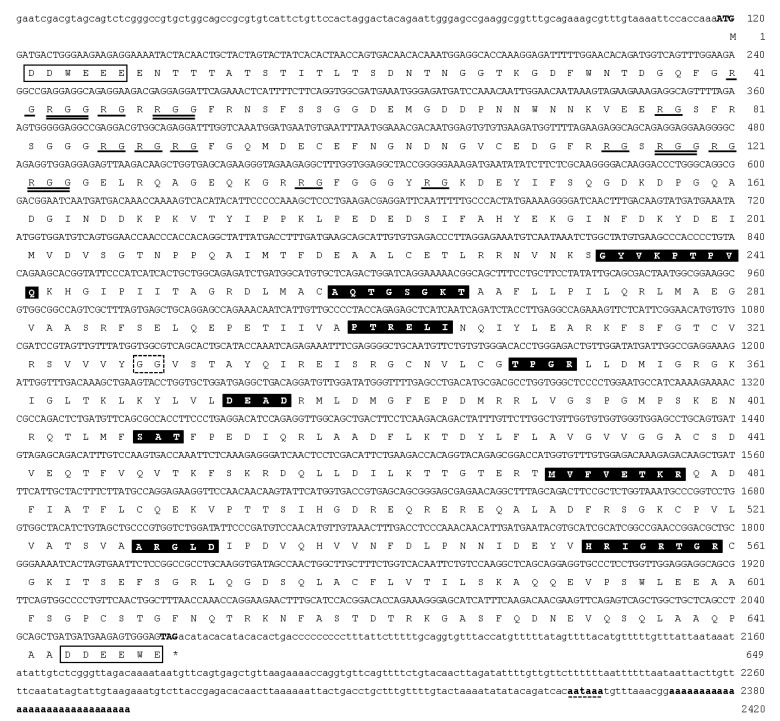
Nucleotide sequence of *ddx4* cDNA and translated amino acid sequence of the encoded protein. White boxes indicate the consensus aspartic acid (D), glutamic acid (E), and tryptophan (W) residues next to the start and stop codons. Arginine–glycine (RG) and arginine–glycine–glycine (RGG) repeats in the N-terminal region are underlined and double-underlined, respectively. Black boxes indicate the nine consensus sequence motifs of DEAD-box proteins, whereas the open box with dotted lines indicates the glycine–glycine (GG) consensus doublet. The predicted polyadenylation signal is underlined with a dotted line. See Table 1 for the primer sequences.

**Figure 2:**
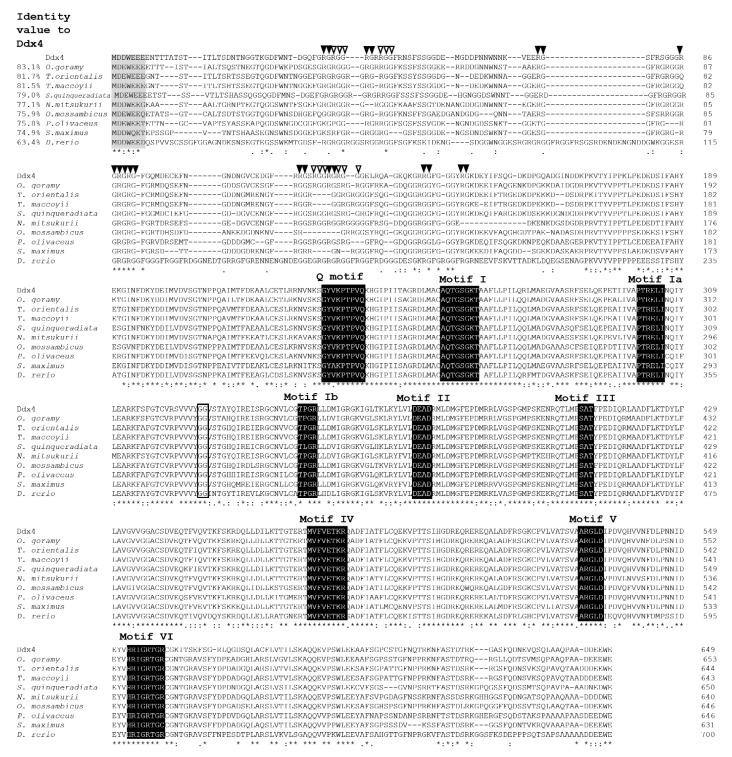
Multiple alignments of the Ddx4 amino acid sequence with known piscine Ddx4 protein homologs. The amino acid sequences were aligned using CLC Main Workbench 7.9.1, and the percentage identities of Ddx4 with the other Ddx4 sequences that had the greatest sequence identity are shown in front of the sequence. Gaps that were introduced to maximize sequence homology are indicated by dashes. Arginine–glycine (RG) and arginine–glycine–glycine (RGG) repeats in the N-terminal regions are indicated by black and white arrowheads, respectively. Shaded boxes indicate the consensus aspartic acid (D), glutamic acid (E), and tryptophan (W) residues next to the start and stop codons. The black boxes show the nine consensus sequence motifs of DEAD-box protein, and the open box represents the glycine–glycine (GG) consensus doublet. Fully, highly, and less conserved amino acid residues are indicated by (*), (:), and (.), respectively. The GenBank Accession numbers of the Ddx4 protein homologs are as follows: *Osphronemus goramy*, GQ422440.1; *Thunnus orientalis*, ABY77970.1; *Thunnus maccoyii*, KP171241.1; *Seriola quinqueradiata*, GU596411.1; *Nibea mitsukurii*, GQ404692.1; *Oreochromis mossambicus*, KR779761.1; *Paralichthys olivaceus*, JQ070418.1; *Scophthalmus maximus*, JX235364.1; *Danio rerio*, BC059794.1.

The full-length cDNA of *ddx4* comprised 2420 base pairs (bp), which included 117 bp of the 5′UTR, 1950 bp of the open reading frame (ORF) encoding 649 amino acid (aa) residues, and 352 bp of the 3′UTR with a poly (A) tail (Figure 1). The deduced amino acid sequence of Ddx4 contained nine consensus motifs of the DEAD-box protein family, including the Q-motif (GYVKPTPVQ), motif I (ATPase-A motif; AQTGSGKT), motif Ia (PTRELI), motif Ib (TPGR), motif II (ATPase-B motif; DEAD), motif III (RNA unwinding motif; SAT), motif IV (MVFVETKR), motif V (ARGLD), motif VI (RNA-binding motif; HRIGRTGR), and a glycine–glycine (GG) doublet. They were rich in glycine residues (26%) between the N-terminus and aa 150, containing nine arginine–glycine (RG) and four arginine–glycine–glycine repeats (RGG), which are conserved among other known Ddx4 proteins. Well-conserved tryptophan (W), glutamic acid (E), and aspartic acid (D) residues were also present near the start and stop codons of Ddx4. Multiple alignments of Ddx4 with other known Ddx4 protein homologs showed that these nine motifs are highly conserved (Figure 2). The deduced aa sequences of Ddx4 had a calculated molecular weight of 15.65 kDa and a theoretical isoelectric point of 4.95. Figure 2 shows the highest identity (63.4–83.1%) of Ddx4 with other fish Ddx4 homolog proteins. Indeed, *ddx4* shared the highest nucleotide identity (data not shown) and deduced amino acids with giant gourami (*Osphronemus goramy*) *ddx4* (Figure 2).

**Figure 3:**
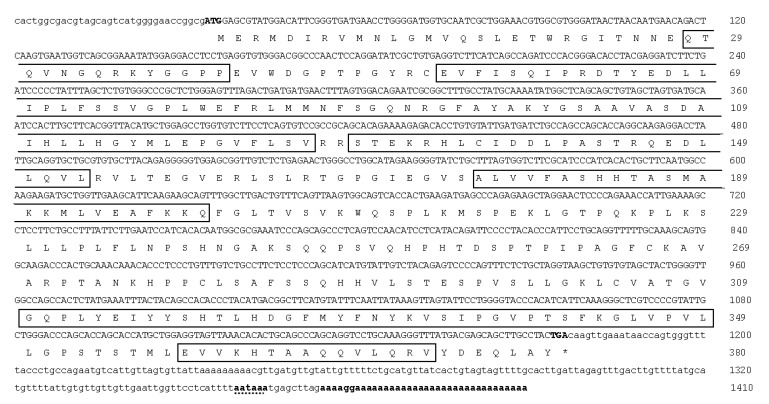
Nucleotide sequence of *dnd1* cDNA and translated amino acid sequence of the encoded protein. White boxes indicate the conserved motifs, including NR, RRM, CR1, CR2, CR3, and CR4. The predicted polyadenylation signal is underlined with a dotted line.

**Figure 4:**
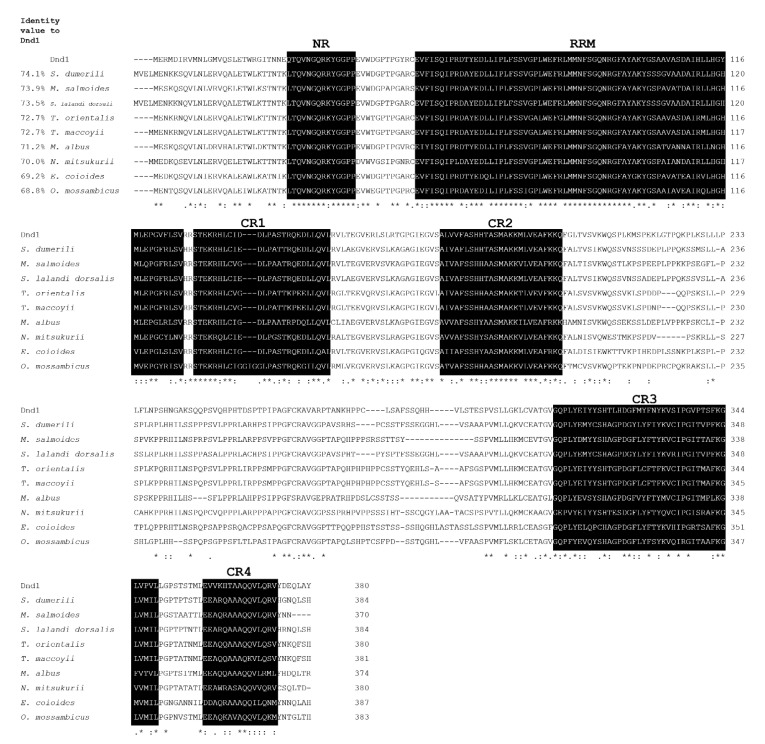
Multiple alignments of the Dnd1 amino acid sequence with known vertebrate protein homologs. The amino acid sequences were aligned using CLC Main Workbench 7.9.1, and the percentage identities of Dnd 1 with the other Dnd sequences that had the greatest sequence identity are shown in front of the sequence. Gaps that were introduced to maximize sequence homology are indicated by dashes. The conserved motifs, including NR, RRM, CR1, CR2, CR3, and CR4, are indicated by black boxes. Fully, highly, and less conserved amino acid residues are indicated by (*), (:), and (.), respectively. The GenBank accession numbers of the Dnd protein homologs are as follows: *Seriola dumerili*, XP_022612856.1; *Micropterus salmoides*, XP_038556163.1; *Seriola lalandi dorsalis*, XP_023273625.1; *Thunnus orientalis*, AHB61249.1; *Thunnus maccoyii*, AKA59813.1; *Monopterus albus*, QBA57712.1; *Nibea mitsukurii*, BBB36995.1; *Epinephelus coioides*, ARQ20732.1; *Oreochromis mossambicus*, ALM89050.1.

The full-length cDNA of *dnd1* contained 1410 bp, which included 34 bp of 5′UTR, 1143 bp of the ORF encoding 380 aa residues, and 233 bp of 3′UTR with a poly (A) tail (Figure 3). The deduced amino acid sequence of the Dnd1 protein contained the typical RNA recognition motif (RRM) and five conserved regions, including an N-terminal region (NR) and four C-terminal regions (CR1-4). Multiple alignments of Dnd1 with other known Dnd protein homologs showed that it is highly conserved for typical RRM, NR, and CR1-4 (Figure 4). The calculated molecular weight and isoelectric point of the deduced aa sequences of Dnd were 12.7 and 9.3 kDa, respectively. Indeed, Dnd1 shared the greatest identity (42.5–66.6%) with the Dnd protein homologs of similar fish species that shared Ddx4, except for *O*. *goramy* (Figure 4).

**Figure 5:**
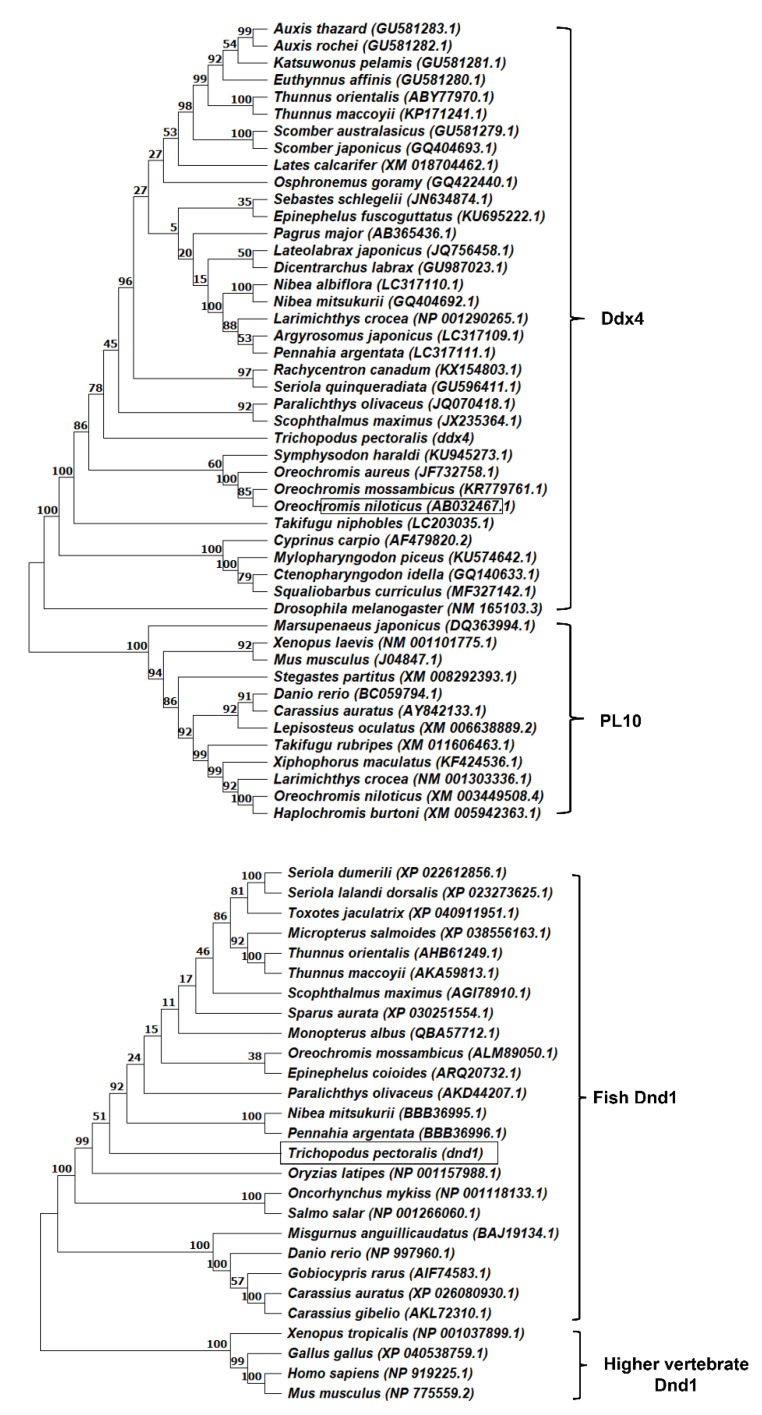
Phylogenetic tree of Ddx4 and PL10 family proteins from different teleost fish species and Dnd from different fish and higher vertebrate. The tree was constructed using 1000 bootstrap replications with MEGA 11 using the UMPGA method [37]. The percentages of replicate trees in which the associated taxa clustered together in the bootstrap test (1000 replicates) are shown above the branches. The GenBank accession numbers of the Ddx4, PL10, and Dnd 1 proteins are provided in brackets.

Phylogenetic tree analysis showed that Ddx4 was clustered in the Ddx4 protein branch (Figure 5). Figure 5 shows that the Ddx4 protein branch was separated from PL10, which also includes DEAD-box RNA helicase. The phylogenetic tree analysis of Dnd1 with other known Dnd protein homologs revealed that the tree was divided into two branches, including the fish branch and that of other vertebrate species, and Dnd1 was located in the fish branch (Figure 5).

### 3.2. Tissue Distribution of ddx4 and dnd1 and In Situ Hybridization 

**Figure 6:**
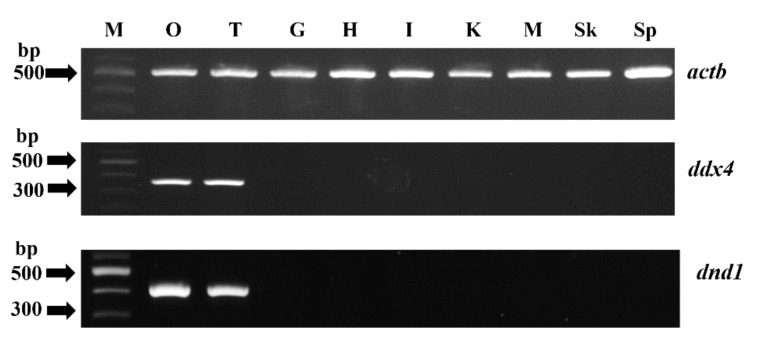
Reverse-transcription polymerase chain reaction (RT-PCR) of *actb*, *ddx4*, and *dnd1* mRNAs in tissues from snakeskin gourami. cDNAs were synthesized using total RNA isolated from the ovary (O), testis (T), gill (G), heart (H), intestine (I), kidney (K), muscle (M), skin (Sk), and spleen (Sp). Distilled water was used as a negative control (data not shown). M represents the DNA marker.

The transcripts of *ddx4* and *dnd1* in various tissues, including the gill, heart, intestine, kidney, liver, muscle, skin, stomach, testis, and ovary, were analyzed using RT-PCR with *actb* as an internal standard. The expressions of both *ddx4* and *dnd1* were detected only in the testis and ovary, whereas that of *actb* was detected in all examined tissues (Figure 6). 

**Figure 7:**
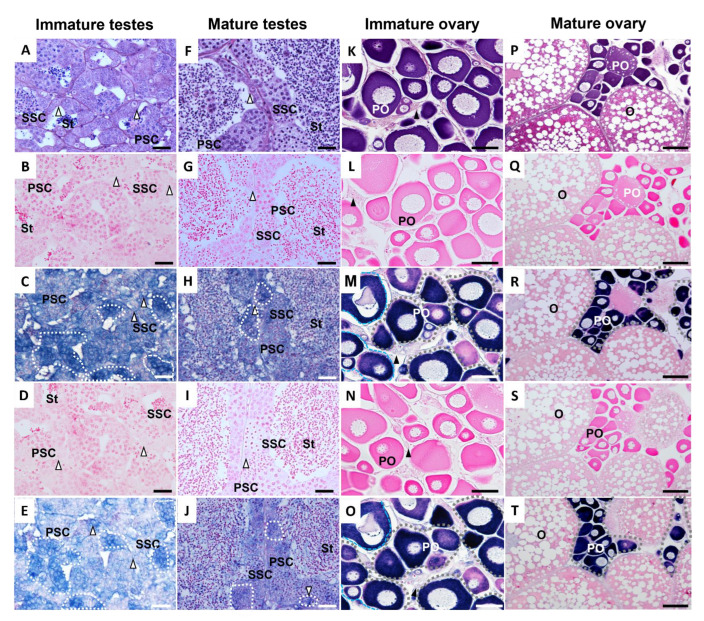
Histological characterization and in situ hybridization using either *ddx4* or *dnd1* antisense probes. Serial transverse sections of an immature testis (**A**–**E**), mature testis (**F**–**J**), immature ovary (**K**–**O**), and mature ovary (**P**–**T**) of snakeskin gourami are shown. The tissues were stained with hematoxylin–eosin (H&E) (**A**,**F**,**K**,**P**) or subjected to in situ hybridization using a sense probe of *ddx4* (**B**,**G**,**L**,**Q**), an antisense probe of *ddx4* (**C**,**H**,**M**,**R**), a sense probe of *dnd1* (**D**,**I**,**N**,**S**), or an antisense probe of *dnd1* (**E**,**J**,**O**,**T**). Scale bars represent 20 µm (**A**–**J**), 50 µm (**K**–**O**), and 100 µm (**P**–**T**). White broken lines encircle positive signals for *ddx4* and *dnd1* in testis. Grey broken lines encircle positive signals for *ddx4* and *dnd1* in ovary. White and black arrowheads indicate spermatogonia and oogonia, respectively. O, maturing oocyte; PO, previtellogenic oocyte; PSC, primary spermatocyte; SSC, secondary spermatocyte; St, spermatid.

Histological studies and in situ hybridization were performed in immature and mature testes and ovaries (Figure 7). The immature testis contained spermatogonia (SG), primary spermatocytes (PSC), secondary spermatocytes (SSC), and small numbers of spermatids (ST) (Figure 7A–E). The immature testis showed strong positive signals for *ddx4* and *dnd1* in SG and reduced signals in PSC and SSC, but no positive signals for ST were observed (Figure 7C,E). All testicular cells, including SG, PSC, SSC, and ST (in high proportions), were observed in the mature testis (Figure 7F–J). Again, positive signals were found in the SG, PSC, and SSC, but not in the ST (Figure 7H,J). No specific signal was detected in immature or mature testes by in situ hybridization with sense *ddx4-*cRNA and *dnd1-*cRNA probes (Figure 7B,D,G,I), indicating the specificity of the antisense *ddx4-*cRNA and *dnd1-*cRNA probes. Immature and mature ovaries contained oogonia (OG) and previtellogenic oocytes (PO) (Figure 7K–O). In addition, mature ovaries continued to mature into oocytes (O). Furthermore, the sense *ddx4-*cRNA and *dnd1-*cRNA probes did not produce specific signals in either the immature or mature ovaries (Figure 7L,N,Q,S). In both the immature and mature ovaries, strong signals for the antisense *ddx4-*cRNA and *dnd1-*cRNA probes were detected in the PO (Figure 7M,O,R,T).

### 3.3. Expression Levels of ddx4 and dnd1 during Early Development

**Figure 8:**
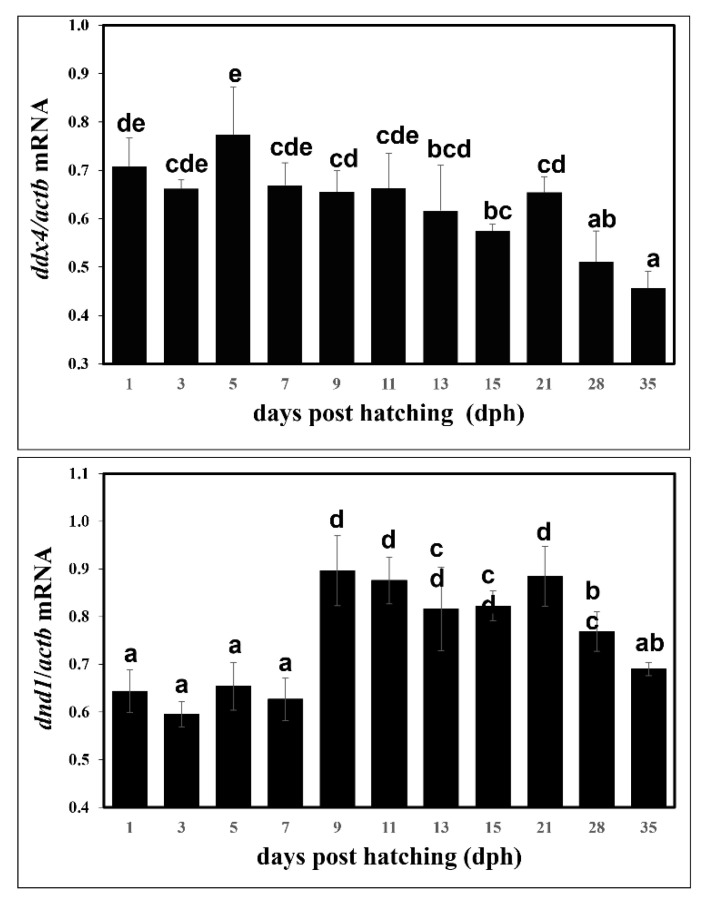
Expression levels of *ddx4* and *dnd1* of snakeskin gourami larvae at various developmental stages. Larva samples were collected at 1, 3, 5, 7, 9, 11, 13, 15, 21, 28, and 35 days post-hatching. The expression level of each gene was normalized with the expression of *actb*. The values are the means + SD from 5 pooled samples (each pooled from 50 mg larval samples) after triplicate PCR analysis. One-way ANOVA followed by Tukey’s range test was performed to rank the treatment groups. Different letters in the bar graph indicate significant differences (*p* < 0.05).

Quantitative RT-PCR was performed to evaluate the relative expression levels of *ddx4* and *dnd1* during larval development at 35 dph. Figure 8A shows that *ddx4* was continuously detectable in larvae at 1–35 dph, and the expression of *ddx4* tended to decrease with increasing developmental age. In addition, *dnd1* was expressed continuously in the larvae at 1–35 dph. However, the expression of *dnd1* increased with increasing developmental age (Figure 8B).

### 3.4. Sexually Dimorphic Expression of ddx4 and dnd1 in Males and Females

**Figure 9:**
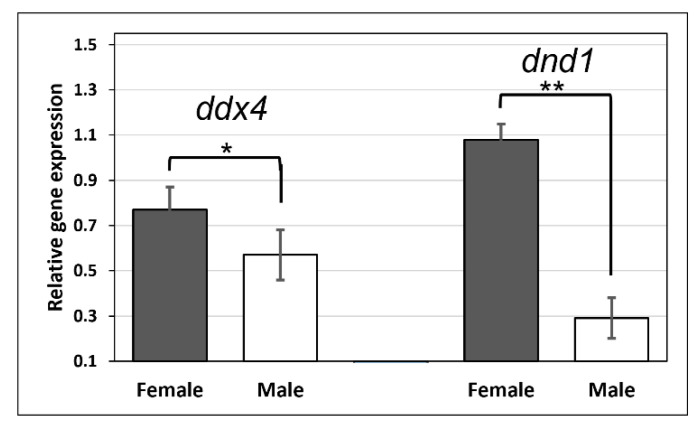
Expression levels of *ddx4* and *dnd1* in ovaries of females and in the testes of males. Gonad samples were collected from snakeskin gourami at the age of 8 months. The relative expression level of each gene was normalized with the expression of *actb*. The values are the means + SD from 5 replicates (2 fish/replicate) after triplicate PCR analysis. An independent *t*-test was performed to determine the sex differences in both *ddx4* and *dnd1* expression. * Significance level at *p* < 0.05; ** Significance level at *p* < 0.001.

Using qRT-PCR, the sexually dimorphic expression levels of *ddx4* and *dnd1* were determined in adult snakeskin gourami (Figure 9). The expression levels of *ddx4* and *dnd1* were higher in the ovaries of females than in the testes of males (Figure 9). 

## 4. Discussion

Several genes that are specifically expressed in the germline have been characterized and used as markers in reproductive studies [6,7,8,9,10,11,12]. Since *ddx4* and *dnd* have been intensively studied for their functions in reproductive biotechnology research and development in aquaculture, in this study, we cloned and characterized *ddx4* and *dnd1* and demonstrated their specific expressions in gonads and various germ cell stages in snakeskin gouramis. Snakeskin gouramis exhibit strong sexual dimorphism in growth; females grow faster, and their body morphology is preferred by consumers over that of their male counterparts. Our findings show the comparative expression levels of *ddx4* and *dnd1* in males and females, which will be useful as sex dimorphic markers in future reproductive studies.

The deduced amino acid sequences of Ddx4 and Dnd1 proteins shared the most consensus motifs with other vertebrate Ddx4 and Dnd families. Ddx4 consists of nine consensus sequence motifs of the DEAD-box protein family, including motifs I, Ia, Ib, II, III, IV, V, VI, and Q motifs. These motifs are essential for binding ATP and RNA and for the hydrolysis of ATP to unwind RNA [24,40,41,42,43]. In addition, the Ddx4 amino acid contains RG and RGG repeats (near the N-terminal regions), which are predicted to be essential for RNA binding [22,44]. Typical consensus aspartate (D), glutamate (E), and tryptophan (W) residues are also located next to the start and stop codons [5]. The phylogenetic analysis demonstrated that Ddx4 clustered with the Ddx4 family and was separated from the PL10 family. For Dnd1, the deduced amino acid sequence of the Dnd1 protein contained the typical RRM and five conserved regions (NR, CR1–4). The RRM is known as the RNA-binding domain necessary for the regulation of protein localization in germ cells [13,45,46,47,48,49]. Phylogenetic analysis demonstrated that Dnd clustered with the Dnd families of teleosts. Both Ddx4 and Dnd1 showed the highest identity with *Thunnus orientalis*, *Thunnus maccoyii*, *Oreochromis mossambicus*, *Paralichthys olivaceus*, *Nibea mitsukurii*, *Scophthalmus maximus*, and *Danio rerio*. Ddx4 and Dnd1 contain essential features of typical Ddx4 and Dnd homologs, respectively.

Using RT-PCR and in situ hybridization, we demonstrated that *ddx4* and *dnd1* are specifically expressed in the gonads, particularly in germ cells. Similar specific expressions of *ddx4* mRNA in the gonads have been demonstrated in rainbow trout [50], medaka [51], tilapia [30], gibel carp [52], rice field eel [53], Pacific bluefin tuna [54], carp [55], catfish [18], European seabass [56], rare minnow [57], Atlantic cod [58], Korean rockfish [59], Solea senegalensis [60], toadfish [61], Japanese flounder [20], brown-marbled grouper [31], striped catfish [7], and Mekong giant catfish [62]. Coincidentally, the specific expression of *dnd1* in testes and ovaries was revealed in zebrafish [15], medaka [29], rare minnow [63], gibel carp [13], tambaqui [64], Atlantic salmon [65], and starry flounder [21]. In addition, in situ hybridization using antisense *ddx4* and *dnd1* probes demonstrated that *ddx4* and *dnd1* mRNAs were observed in germ cells at various stages, and strong expression was observed in immature germ cells in testes, including spermatogonia, PSC, and SSC, and in ovarian cells such as oogonia and primary oocytes. Our findings are consistent with those of previous studies conducted on teleost fish [11,19,29,31,52,59,62,64]. *ddx4* and *dnd1*, which revealed consensus sequences, sequence homology, and phylogenetic analysis with other teleost *ddx4* and *dnd* homologs, exhibited germline-specific expressions, suggesting that the characteristics of *ddx4* and *dnd1* homologs can be used as germline markers for further investigation of gonadal development in snakeskin gourami.

Ontogenetic variations in the level of *ddx4* mRNA in several fish species have demonstrated that maternal transcripts of *ddx4* persist, and their amounts have been shown to be relatively high at the early developmental stage and diluted in later embryogenesis [18,20,51,52]. A similar ontogenetic expression pattern of dnd during embryo development has also been revealed in a number of fish [9,11,15,19,21,29,65,66]. The present study determined the expression levels of *ddx4* and *dnd1*, which have different patterns, from hatching through to early larval development at 35 dph (1 cm). Note that the total RNA was extracted from the whole bodies of fry because the fry were too small to isolate the gonads, and the transcripts of *ddx4* and *dnd1* were diluted with the content of other gene transcripts in the growing fry. The expression of *ddx4* was high during early larval development and decreased with increasing developmental age. A similar trend of the expression level of *ddx4* was previously observed in European sea bass [56]. However, an inverse expression pattern was observed for *dnd1*, where an increase in *dnd1* was observed with increasing developmental age. These findings suggest different roles for *ddx4* and *dnd1*, although both genes are essential for the regulation of early gonadal development in vertebrates [14,15,29,67]. Indeed, sex differentiation in snakeskin gourami is not completed 52 d post-hatching (data not shown). Therefore, a developmental age of 35 dph would correspond to the migration and subsequent proliferation of primordial germ cells in snakeskin gourami, and the upregulation of *dnd1* is crucial for the processes of early gonadal development. In medaka, *ddx4* is essential for the migration of PGC, but not for PGC proliferation and survival [68]. In zebrafish and orange-spotted groupers, *dnd* is essential for PGC migration and survival [11,15]. *dnd* knockdown using antisense morpholino and *dnd* knockout have been applied as tools to produce PGC-depleted gonads in zebrafish and Atlantic salmon [27,49]. 

This study demonstrated the sexually dimorphic expression levels of *ddx4* and *dnd1* during the adult stage. The qRT-PCR analysis showed that in the adult snakeskin gourami, the expressions of *ddx4* and *dnd1* were higher in the ovary than in the testis. Similarly, higher expression in the ovary than in the testis was demonstrated for *ddx4* in European seabass (*Dicentrarchus labrax*) [56], Japanese flounder (*Paralichthys olivaceus*) [20], and catfish [18], and for *dnd* in starry flounder *(Platichthys stellatus*) [21] and olive flounder (*Paralichthys olivaceus*) [9]. However, similar levels of *ddx4* expression between the ovary and testis have been demonstrated in rare minnow [57]. In the hermaphrodite orange-spotted grouper, higher expressions of *dnd* were reported in mature testes than in mature ovaries, and its expression level changed during sex reversal [11]. The sexually dimorphic expression of *dnd* varies depending on gonadal development in turbot (*Scophthalmus maximus*) [19]. In addition, differential expressions of *ddx4* vary according to the different stages of the seasonal reproductive cycle in the testes and ovaries of catfish [18]. Therefore, the sexual dimorphism of *ddx4* and *dnd* expression depends on the species, reproductive system, developmental age, and/or gonadal development. 

## 5. Conclusions

In summary, *ddx4* and *dnd1* homologs were cloned and characterized from snakeskin gourami, indicating their potential further use as germline DNA markers. They were specifically expressed in the gonads, and their strong expression was detected in immature germ cells. Both *ddx4* and *dnd1* were expressed during early gonadal development in fry; however, their expression levels showed opposite trends. Sexually dimorphic expressions of *ddx4* and *dnd1* in adult snakeskin gourami were revealed.

## Figures and Tables

**Table 1:** The oligonucleotide primers used in this study.

Primer Name	5′ to 3′ Nucleotide Sequences	Purposes
Tpe-dndF1	AAYGGNCARMGNAARTAYGG	Cloning, RT-PCR
Tpe-dndR1	TGNCCNSWRAARTTCATCAT	Cloning
Tpe-dndF2	CTGATCCCCCTATTTAGCTCTGTG	RACE PCR
Tpe-dndR2	CACAGAGCTAAATAGGGGGATCAG	RACE PCR
Tpe-dndF3	GCTCTGGGAGTTTAGACTGATGATG	In situ hybridization
Tpe-dndR3	CATGAAGCCGTCATGTAGGGTGTG	In situ hybridization
Tpe-dndF4	GCTGTGAGGTCTTCATCAGCCAGA	qRT-PCR
Tpe-dndR4	TCCAGCATGTAACCGTGAAGCAAG	qRT-PCR, RT-PCR
Tpe-ddx4F1	GAYGABATMHTKGTVGAYGTBAGYGG	Cloning
Tpe-ddx4F2	AAGCCBACYCCDGTVCAGAARYAYGG	Cloning, in situ hybridization, RT-PCR
Tpe-ddx4R1	CCABKWVGGMACYTCCTGYTGRGG	Cloning
Tpe-ddx4R2	TTHCCRCAKCGDCCDGTKCKBCCRA	Cloning, in situ hybridization
Tpe-ddx4R3	AGTCGCTGCAATATAGGAAGCAGG	RACE PCR, RT-PCR
Tpe-ddx4R4	CAGGTGTCCCACACAGAACATTGC	RACE PCR
Tpe-ddx4F3	AGGTTGGCAGCTGACTTCCTCAAG	RACE PCR
β-actinF	ACTACC TCA AGATCCTG	RT-PCR
β-actinR	TTGCTGATCCACATCTGCTG	RT-PCR
Tpe-ddx4F4	AGTCGCTTTAGTGAGCTGCAGGAG	qRT-PCR
Tpe-ddx4R5	CTGATTTGGTATGCAGTGCTGACG	

## Data Availability

Not applicable.

## Supplementary Materials

The following supporting information can be downloaded at: https://www.mdpi.com/article/10.3390/ani12233415/s1.
