# Supplementary material for: Characterization of ddx4 and dnd Homologs in Snakeskin Gourami (Trichopodus pectoralis) and Their Expression Levels during Larval Development and in Gonads of Males and Females
Source: Animals (Basel). 2022 Dec 4;12(23):3415. doi: 10.3390/ani12233415 (PMC9735842; doi:10.3390/ani12233415)
Supplement: Supplementary file 1 [file animals-12-03415-s001.zip › animals-1973191-supplementary.pdf]

Supplementary Information I

S1A. Multiple alignment of amino acid of the known piscine Ddx4 protein homologs and location of degenerate primer design. *C. carpio*, AF479820.2; *L. japonicus*, JQ756458.1; *E. fuscoguttatus*, KU695222.1;*D. labrax*, GU987023.1; *T. orientalis*, ABY77970.1; *T. maccoyii*, KP171241.1; *O, goramy*, GQ422440.1; *L. calcarifer*, XM_018704462.1

```
C.carpio            MDDWEEDQGPVVSCSSGFGLSSRGADGGFKSFYTGSTGNGKANNEDGDGGSWKTTAGDGF     60
D.labrax            MDEWEEEGTTITSTTT---L------------------TT--TSEGRQEGSWNTANGESG     37
E.fuscoguttatus     MDEWEEEGTTTST-----------------------------IAPTSQGDTWNTGGGEFS     31
T.orientalis        MDEWEEEGN--TSTIT---L------------------TSRTSSEGTQGDFWNTNGGEFG     37
T.maccoyii          MDEWEEEGN--TSTIT---L------------------TSHTSSEGTQGDFWNTNGGEFG     37
O.goramy            MDEWEEEETTTISTIA---L------------------TSQSTNEGTQGDFWKPDSGESG     39
L.japonicus         MDEWEEEGT-PTSTIT---L------------------TSHTSSE---------------     23
L.calcarifer        MDDWEEET--ATSPIT---L------------------TSHTSNEVTQGDSWNAGGGEFG     37
                    **:***:     :

C.carpio            RGRGGRGGSRGGRGGFRNTFKSETDENGNDE-GWKNGEGQGRGRGGFRGGFRGGFRGGFR    119
D.labrax            RGRGGRG--R--GGGFKSSFSSD-------GDNWNSTAG----EGGFRG-----------     71
E.fuscoguttatus     RGRGGRG--RGRQGGFKSSFSSGGDENGNDQDTWNHAGGD---RGGFRG-----------     75
T.orientalis        RGRGGRG--RGR-GGFKSSYSSGGDG--NDEDKWNNAGGE---RGGFRG-----------     78
T.maccoyii          RGRGGRG--RGR-GGFKSSYSSGGDG--NDEDKWNNAGGE---RGGFRG-----------     78
O.goramy            RGRGGGG--RGRRGGFKSSFSSGGEERRDDGNNWNSTAAE---RGGFRG-----------     83
L.japonicus         ---GCGG--RGRRGGFKSLFSSGGDENQNDGDNWNNTGGE---RGGFRG-----------     64
L.calcarifer        GGRGGRG--KGRRGGFTSSFSG----DENGNDSWNNTGGE---RGGFRD-----------     77
                       *  *  :   *** . :..           *:   .    .****.

C.carpio            EGGNEETGKKGFGREND-------GNGTDGGWKGGEGRGIGRGGFRGGFRDGSGDEETGK    172
D.labrax            -RGG-RGRGRGFSRTDRSEFNEDDNQVCENG--GGSRGGRG---GRGGFRSG--GDQ-GC    121
E.fuscoguttatus     -RGGRRGRGRGFGGTDGSEFSGDDNGVCENGFRGGSRGGR----GGRGFRQG--GDQ-GG    127
T.orientalis        -RGG-QGRGRGFCRMDQSEFNGDDNGMRENGYRGGRG-GRG---RGGGFSQG--GDQ-GG    129
T.maccoyii          -RGG-QGRGRGFCRMDQSEFNGDDNGMRENGYRGGRG-GRG---RGGGFSQG--GDQ-GG    129
O.goramy            -RGG-RGRGRGFGRMDQSEFNGDDSGVCESGFRGGSRGGRGSR-GRGGFREA--GDQ-GG    137
L.japonicus         -RGG-RGRGRGFDRRDRSEFNENNDGVCENGFRGGSRGGRGGR-GG-GFRQG--GDQ-GG    117
L.calcarifer        -RGG-RGRGRGFCRTDPSEFTGDDDGVCENGFRGGSRGGRGSR-GGRGFRQG--GER-GG    131
                      *. .   :**   :        .   :.*  **   *        ** ..  .:. *

C.carpio            RGFGRGGFRGRNEEVFSKASTADKPDPEAGENAGPKVVYVPPPPPEEESSIFSHYASGIN    232
D.labrax            RGGFGGGYRGKDEEVFSQREDKE--NKDATDGERPRVTYIPPTLPEDEDSIFAHYETGIN    179
E.fuscoguttatus     RGGFGGGYRGKDEEVFAKGDDRDPE-KDAGDGDKPKVTYIPPSLPDDEDSIFAHYESGIN    186
T.orientalis        RGGFGGGYRGKDEEIFTRGEDKDPEKKD--DSDRPKITYVPPTLPEDEDSIFSHYETGIN    187
T.maccoyii          RGGFGGGYRGKE-EIFTRGEDKDPEKKD--DSDRPKITYVPPTLPEDEDSIFSHYETGIN    186
O.goramy            RGGYGGGYRGKDEEIFAQGENKDPGKKDAIDGDRPKVTYVPPTLPEDEDSIFAHYKTGIN    197
L.japonicus         KGGFGGGYRGKDEEIFAQRGEDKEK-KDASDGDRPKVTYVPPTLPEDEDSIFSHYETGIN    176
L.calcarifer        RGGFGGGYRGKDEQVFAQGEDKDPG-NDASEGDRPKITYIPPTLPEDEDAVFAHYETGIN    190
                    :*   **:**:: ::*::    .    :  :.  *::.*:**  *::*.::*:** :***

                        Tpe-ddx4F1                          Tpe-ddx4F2
C.carpio            FDKYDDILVDVSGSNPPKAIMTFDEAGLCESLRKNVTKSGYVKPTPVQKHGIPIISAGRD    292
D.labrax            FDKYDDILVDVSGANPPQAIMTFDEAGLCESLRKTVSKSGYVKPTPVQKHGIPIISAGRD    239
E.fuscoguttatus     FDKYDDIMVDVSGTNPPQAIMTFDEAALCESLRKNIKKSGYVKPTPVQKYGIPIISAGRD    246
T.orientalis        FDKYDDIMVDVSGTNPPQAVMTFDEAALCESLRKNVSKSGYVKPTPVQKHGIPIISAGRD    247
T.maccoyii          FDKYDDIMVDVSGTNPPQAVMTFDEAALCESLRKNVSKSGYVKPTPVQKHGIPIISAGRD    246
O.goramy            FDKYDDIMVDVSGTNPPQAILTFDEAALCETLRKNVSKSGYVKPTPVQKHGIPIISAGRD    257
L.japonicus         FDKYDDIMVDVSGTNPPQAIMTFDEAALCESLRKNVSKSGYVKPTPVQKHGIPIISAGRD    236
L.calcarifer        FDKYDDIMVDVSGTNPPEALMTFDEAMLCESLRKNVSKSGYVKPTPVQKHGIPIISAGRD    250
                    *******:*****:***:*::***** ***:***.:.************:**********

C.carpio            LMACAQTGSGKTAAFLLPILQRFMTDGVAASKFSEIQEPEAIIVAPTRELINQIYLEARK    352
D.labrax            LMACAQTGSGKTAAFLLPILQQLMADGVAASSFSELQEPEAVIVAPTRELINQIYLEARK    299
E.fuscoguttatus     LMACAQTGSGKTAAFLLPILQQLMADGVAASSFSELQEPETIIVAPTRELINQIFLEARK    306
T.orientalis        LMACAQTGSGKTAAFLLPILQQLMADGVAASRFSELQEPEAIIVAPTRELINQIYLEARK    307
T.maccoyii          LMACAQTGSGKTAAFLLPILQQLMADGVAASRFSELQEPEAIIVAPTRELINQIYLEARK    306
O.goramy            LMACAQTGSGKTAAFLLPILQQLMADGVAASRFSELQEPEALIVAPTRELINQIYLEARK    317
L.japonicus         LMACAQTGSGKTAAFLLPILQQLMADGVAASRFSELQEPEAIIVAPTRELINQIYLEARK    296
L.calcarifer        LMACAQTGSGKTAAFLLPILQQLMADGVAASRFSELQEPEAIIVAPTRELINQIYLEARK    310
                    *********************::*:****** ***:****::************:*****
```

```
C.carpio           FAYGTCVRPVVVYGGINTGYTIREVLKGCNVLCATPGRLHDLIGRGKIGLSKVRYLVLDE    412
D.labrax           FAFGTCVRPVVVYGGVSTGHQIREISRGCNVLCGTPGRLLDVIGRGKVGLSKVRYFVLDE    359
E.fuscoguttatus    FAFGTCVRPVVVYGGVSTGHQIREICRGCNVVCGTPGRLLDVIGRGKIGLSKLRYLVLDE    366
T.orientalis       FAFGTCVRPVVVYGGVSTGHQIREIERGCNVVCGTPGRLLDMIGRGKVGLSKLRYLVLDE    367
T.maccoyii         FAFGTCVRPVVVYGGVSTGHQIREIERGCNVVCGTPGRLLDMIGRGKVGLSKLRYLVLDE    366
O.goramy           FSFGTCVRPVVVYGGVSTAHQIREISRGCNVLCGTPGRLLDVIGRGKVGLSKLRYLVLDE    377
L.japonicus        FAFGTCVCPVVVYGGASTGHQIREISRGCNVLCGTPGRLLDMIGRGKVGLSKLRYFVLDE    356
L.calcarifer       FAFGTCVRPVVVYGGVSTGYQIREISRGCNVLCGTPGRLLDMIGRGKVGLQKLRYLVLDE    370
                   *::**** ******* .*.: ***: :****:*.***** *:*****:**.*:**:****

C.carpio           ADRMLDMDMGFEPDMRKLVGSPGMPSKEDRQTLMFSATYPEDIQRMAADFLKVDYIFLAV    472
D.labrax           ADRMLD--MGFEPDMRRLVGSPGMPSKENRQTLMFSATYPEDIQRMAADFLKTDYLFLAV    417
E.fuscoguttatus    ADRMLD--MGFEPDMRRLVGSPGMPPKESRQTLMFSATYPEDIQRMAADFLKTDYLFLAV    424
T.orientalis       ADRMLD--MGFEPDMRRLVGSPGMPSKENRQTLMFSATYPEDIQRMAADFLKTDYLFLAV    425
T.maccoyii         ADRMLD--MGFEPDMRRLVGSPGMPSKENRQTLMFSATYPEDIQRMAADFLKTDYLFLAV    424
O.goramy           ADRMLD--MGFEPDMRRLVGSPGMPSKENRQTLMFSATYPEDIQRMAADFLKTDYLFLAV    435
L.japonicus        ADRMLD--MGFEPDMRRLVGSPGMPSKENRQTLMFSATYPEDIQRMAADFLKTDYLFLAV    414
L.calcarifer       ADRMLD--MGFEPDMRRLVGSPGMPSKEDRQTLMFSATYPEDIQRMAADFLKTDYLFLAV    428
                   ******  ********:******** **.*********************.**:****

C.carpio           GVVGGACSDVEQTIVQVTS-TKRDQLLELLRSTGNERTMVFVETKRSADFIATFLCQEKM    531
D.labrax           GVVGGACSDVEQTFVQVTKFSKREQLLDLLKTTGMERTMVFVETKRQADFIATYLCQEKV    477
E.fuscoguttatus    GIVGGACSDVEQSFVQVTKFSKREQLLDLLKTTGTERTMVFVETKRQADFIATYLCQEKV    484
T.orientalis       GVVGGACSDVEQTFIQVTKFSKREQLLDLLKTTGTERTMVFVETKRQADFIATFLCQEKV    485
T.maccoyii         GVVGGACSDVEQTFIQVTKFSKREQLLDLLKTTGTERTMVFVETKRQADFIATFLCQEKV    484
O.goramy           GVVGGACSDVEQTFVQVTKFSKREQLLDLLKTTGTERTMVFVETKRQADFIATFLCQEKV    495
L.japonicus        GVVGGACSDVEQTFVQVTKFSKREQLLDLLKSTGMERTMVFVETKRQADFIATYLCQEKV    474
L.calcarifer       GVVGGACSDVEQTFIEVTKFSKREQLLDLLKTTGTERTMVFVETKRQADFIATFLCQEKV    488
                   *:*********::::**. :**:***:**::** **********.*****:*****:

C.carpio           STTSIHGDREQREREKALSDFRTGQCPVLVATSVAARGLDIEQVQHVVNFDLPSSIDEYV    591
D.labrax           PTTSIHGDREQREREQALADFRSGKCPVLVATSVAARGLDIPDVQHVVNFDLPNNIDEYV    537
E.fuscoguttatus    PTTSIHGDREQREREQALADFRSGKCPVLVATSVAARGLDIPDVQHVVNFDLPNNIDEYV    544
T.orientalis       PTTSIHGDREQREREQALADFRSGKCPVLVATSVAARGLDIPDVQHVVNFDLPNNIDEYV    545
T.maccoyii         PTTSIHGDREQREREQALADFRSGKCPVLVATSVAARGLDIPDVQHVVNFDLPNNIDEYV    544
O.goramy           PTTSIHGDREQREREQALADFRSGKCPVLVATSVAARGLDIPDVQHVVNFDLPSNIDEYV    555
L.japonicus        PTTSIHGDREQREREQALADFRSGKCPVLVATSVAARGLDIPDVQHVVNFDLPNNIDEYV    534
L.calcarifer       PTTSIHGDREQREREQALADFRSGKCPVLVATSVAARGLDIPDVQHVVNFDLPNNIDEYV    548
                    *************:**:***:*:**************** :**********..*****

                      Tpe-ddx4R2                            Tpe-ddx4R1
C.carpio           HRIGRTGRCGNIGRAVSFFNPESDTPLARSLVKVLSGAQQEVPKWLEEIAFSAHG-TTGF    650
D.labrax           HRIGRTGRCGNTGRAVSFFDPDSDDQLARSLVTILSKAQQEVPSWLEDSAFSGPGSM--G    595
E.fuscoguttatus    HRIGRTGRCGNTGRAVSFYDPDNDNQLARSLVTILSKAQQEVPAWLEESAFSGGGGGSGF    604
T.orientalis       HRIGRTGRCGNTGRAVSFYDPDADGQLARSLVTVLSKAQQEVPSWLEESAFSGP-ATTGF    604
T.maccoyii         HRIGRTGRCGNTGRAVSFYDPDADGQLARSLVTVLSKAQQEVPSWLEESAFSGP-ATTGF    603
O.goramy           HRIGRTGRCGNTGRAVSFYDPEADGHLARSLVGVLSKAQQEVPSWLEEAAFSGP-SSTGF    614
L.japonicus        HRIGRTGRCGNTGRAVSFYDPDADGQLARSLVTVLSKAQQEVPSWLEEFAFSGP-ATTGF    593
L.calcarifer       HRIGRTGRCGNVGRAVSFYDPDADVQLARSLVTVLSKAQQVVPSWLEEHAFSGL--STGF    606
                   *********** ******::*: *  ****** :** *** ** ***: ***.

C.carpio           NPRGKVFASTDTRKG---GSFKTDEPPQASAPSPAAAADDEEWE  691
D.labrax           VAPRKTFASSDSRKGPQGGSVQDDSV-QSQSAAQTAH-DEEEWD  637
E.fuscoguttatus    NAPRKSFASTDSRK---GGSSQDNSV-KSPPAAQSAN-DDEEWE  643
T.orientalis       NPPRKNFASTDSRK---RGSFQDNSV-KSQPAVQTAADDDEEWE  644
T.maccoyii         NPPRKNFASTDSRK---RGSFQDNSV-KSQPAVQTAADDDEEWE  643
O.goramy           NPPRKNFASTDTRQ---RGLLQDTSV-MSQPAAQPAA-DEEEWE  653
L.japonicus        NPPRKNFASSDSRK---GGSFQDNGM-ESLPAAQAAA-DDEEWE  632
L.calcarifer       NPPRKNFASTDSRK---GGSFQDNMM-KSQPAALAAA-DEEEWE  645
                       * ***:*:*:    *  :      :      *  *:***:
```

S1B. Nucleotide alignment of the known piscine ddx4 homologs degenerate oligonucleotide primer design for cloning of *ddx4. C. carpio*, AF479820.2; *L. japonicus*, JQ756458.1; *E. fuscoguttatus*, KU695222.1;*D. labrax*, GU987023.1; *T. orientalis*, ABY77970.1; *T. maccoyii*, KP171241.1; *O, goramy*, GQ422440.1; *L. calcarifer*, XM_018704462.1

```
D.labrax             ------------------------------------------------------------
L.japonicus          ------------------------------------------------------------
T.orientalis         ------------------------------------------------------------
T.maccoyii           ------------------------------------------------------------
O.goramy             ------------------------------------------------------------
L.calcarifer         ------------------------------------------------------------
E.fuscoguttatus      ------------------------------------------------------------
C.carpio             ATGGATGACTGGGAGGAAGATCAGGGCCCTGTTGTATCCTGCAGTTCAGGTTTTGGTCTG

D.labrax             ----ATGGATGAGTGGGAAGAAGAGGGAACTACTATTACTAGCACTACCACACTAACCAC
L.japonicus          ----ATGGACGAATGGGAAGAAGAAGGAACTCC---TACAAGTACTATAACACTAACCAG
T.orientalis         ----ATGGATGAGTGGGAAGAAGAGGGAAATACT------AGCACTATTACACTAACCAG
T.maccoyii           ----ATGGATGAGTGGGAAGAAGAGGGAAATACT------AGCACTATTACACTAACCAG
O.goramy             ----ATGGATGAGTGGGAAGAAGAGGAAACTACTACTATTAGTACTATCGCACTAACCAG
L.calcarifer         ----ATGGATGATTGGGATGAAGAGGCAACTGCT---ACCAGTGCTATTACACTAACCAG
E.fuscoguttatus      ----ATGGACGAGTGGGAAGAAGAGGGAACTACC---ACTAGTACTATTGCACCAACCAG
C.carpio             AGCTCTAGAGGTGCAGACGGAGGATTCAAGAGCTTTTA-TACAGGAAGTACAGGAAACGG
                          * ** *    *   ** **   **   *       *     *   **  ** *

D.labrax             CACT------AGTGAAGGCAGACAAGAAGGGTCTTGGAATAC---TGCCAATGGTGAATC
L.japonicus          CCAC------ACCT----CAAGTGAAGGTTGTGGTGGAAGAG----GCAGACGAGGAGGA
T.orientalis         CCGCACCTCAAGTGAAGGCACACAAGGAGACTTCTGGAACAC---CAATGGTGGTGAATT
T.maccoyii           CCACACCTCAAGTGAAGGCACACAAGGAGACTTCTGGAACAC---CAATGGTGGTGAATT
O.goramy             TCAAAGCACAAATGAAGGCACTCAAGGAGATTTCTGGAAACC---AGACAGCGGTGAATC
L.calcarifer         CCACACCTCAAATGAAGTCACACAAGGAGACTCATGGAACGC---TGGTGGTGGTGAATT
E.fuscoguttatus      C---------------------CAAGGAGATACCTGGAACAC---TGGTGGTGGAGAATT
C.carpio             GAAGGCAAACAATGAAGATGGTGATGGTGGCTCATGGAAGACGACTGCTGGTGATGGATT
                                                       *****             *  *

D.labrax             T---GGAAGGGGTCGTGGTGGAAGGGGCAGAGG------AGGAGGATTTAAAAGCTCATT
L.japonicus          T---TTAAAAGCTTGTTCTCTTCAGG--TGGTG------ACGAGAATCAGAA--------
T.orientalis         T---GGAAGGGGTCGCGGTGGAAGAGGCAGAGGGAG---AGGAGGATTTAAAAGCTCATA
T.maccoyii           T---GGAAGAGGTCGCGGTGGAAGAGGCAGAGGGAG---AGGAGGATTTAAAAGCTCATA
O.goramy             T---GGAAGGGGTCGTGGAGGTGGAGGCAGAGGAAGACGAGGAGGATTCAAAAGCTCATT
L.calcarifer         T---GGAGGGGGTCGCGGTGGAAGAGGCAAAGGCAGACGAGGGGGATTTACAAGCTCATT
E.fuscoguttatus      T---AGCAGGGGTCGTGGTGGAAGAGGCAGAGGCAGACAAGGAGGATTTAAAAGCTCGTT
C.carpio             CAGGGGCAGAGGAGGCAGGGGAGGATCACGAGGAGGAAGAGGAGGCTTCAGAAATACCTT
                               *   *                 *      * * *  *    *

D.labrax             CTCCTC---------------------AGATGGCGACAACTGGAACAGTACAGCTGGAGA
L.japonicus          ---------------------------TGATGGGGACAACTGGAACAATACAGGTGGAGA
T.orientalis         CTCCTCAGGTGGAGATGGGA------ATGATGAGGACAAATGGAACAATGCAGGAGGAGA
T.maccoyii           CTCCTCAGGTGGAGATGGGA------ATGATGAGGACAAATGGAACAATGCAGGAGGAGA
O.goramy             CTCCTCAGGAGGTGAGGAGAGAAGGGATGATGGGAACAACTGGAACAGTACAGCAGCAGA
L.calcarifer         CTC------AGGTGATGAGA------ATGGGAATGACAGCTGGAACAATACAGGAGGGGA
E.fuscoguttatus      CTCCTCAGGTGGTGATGAGAATGGGAACGATCAGGACACTTGGAACCATGCAGGAGGAGA
C.carpio             TAAATCAGAAACTGATGAAAATGGCAACGATGAGGGTTGGAAAAATGGTGAAGGCCAAGG
                                                 *              **    *  **      *

D.labrax             AGGAGGT---------TTTAGAGGTAGA------GGAGG---CAGAGGGCGTGGCAGAGG
L.japonicus          AAGAGGT------GGTTTCAGAGGTAGA------GGAGG---CAGAGGGCGTGGCAGGGG
T.orientalis         AAGAGGT------GGTTTCAGAGGTAGA------GGAGG---CCAAGGGCGCGGCAGAGG
T.maccoyii           AAGAGGT------GGTTTCAGAGGTAGA------GGAGG---CCAAGGGCGCGGCAGAGG
O.goramy             AAGAGGT------GGTTTCAGAGGTAGA------GGAGG---CCGAGGTCGTGGCAGAGG
L.calcarifer         AAGAGGT------GGTTTCAGAGATAGA------GGCGG---TCGAGGGCGTGGCAGAGG
E.fuscoguttatus      CAGAGGT------GGTTTCAGAGGGAGA------GGAGGACGCAGAGGGCGCGGCAGAGG
C.carpio             AAGAGGTCGTGGTGGTTTCCGTGGTGGTTTCCGCGGGGGTTTTCGCGGTGGTTTCCGTGA
                       *****         **   * *   *       ** **       **   *   * * *
```

```
D.labrax            ATTCAGCC--GAACGGATCGCAGTGAA----TTCAATGAGGATGACAATCAAGTGTGTGA
L.japonicus         ATTTGACA--GAAGGGATCGCAGTGAA----TTCAATGAAAACAATGATGGAGTGTGTGA
T.orientalis        ATTTTGCA--GAATGGATCAAAGTGAA----TTCAATGGAGATGACAATGGAATGCGTGA
T.maccoyii          ATTTTGCA--GAATGGATCAAAGTGAA----TTCAATGGAGATGACAATGGAATGCGTGA
O.goramy            ATTTGGTC--GAATGGATCAGAGTGAA----TTCAACGGTGATGACAGTGGAGTGTGTGA
L.calcarifer        ATTTTGCA--GAACGGATCCGAGTGAA----TTCACTGGAGATGACGATGGAGTGTGTGA
E.fuscoguttatus     ATTTGGCG--GGACGGATGGCAGTGAA----TTCAGTGGAGATGATAATGGAGTGTGTGA
C.carpio            AGGTGGCAATGAAGAGACTGGGAAAAAAGGCTTTGGAAGAGAAAATGACGGAAATGGCAC
                    *         * *  **        **    **        *  *      *    *

D.labrax            AAATGG------GGGAGGCAGCCGAGGCGGCAGAGGAGGCAGAGGAGG------------
L.japonicus         AAATGGCTTTAGGGGAGGAAGCAGAGGAGGAAGAGGTGGCAGAGGAGGA------GG---
T.orientalis        AAATGGGT---ATAGAGGAGGAAGAGGGGGCAGAGGAAGAGGAGGAGG------------
T.maccoyii          AAATGGGT---ATAGAGGAGGAAGAGGGGGCAGAGGAAGAGGAGGAGG------------
O.goramy            AAGTGGGTTTAGAGGAGGAAGCCGAGGTGGAAGAGGCAGCAGAGGAAGA------GGAGG
L.calcarifer        AAATGGGTTTAGAGGAGGAAGCCGAGGAGGAAGAGGCAGCAGAGGAGGT------CGAGG
E.fuscoguttatus     AAATGGGTTTAGAGGAGGAAGCCGAGGAGGAAGAGGCGGCAGAGG---------------
C.carpio            AGATGGGGGTTGGAAAGGAGGTGAAGGCCGAGGAATAGGTCGTGGAGGCTTCCGTGGGGG
                    *  ***         ***  *   ***  *  **    *  * **

D.labrax            TTTCAGATCAGGTGGTGAC--CAAGGTTGCAG----AGGAGGATTTGG---AGGAGGCTA
L.japonicus         TTTCAGACAAGGTGGTGAC--CAGGGTGGCAA----AGGAGGCTTTGG---AGGAGGCTA
T.orientalis        TTTCAGCCAAGGTGGCGAC--CAGGGTGGCAG----AGGAGGCTTTGG---AGGAGGTTA
T.maccoyii          TTTCAGCCAAGGTGGCGAC--CAGGGTGGCAG----AGGAGGCTTTGG---AGGAGGTTA
O.goramy            CTTCAGAGAAGCTGGTGAC--CAGGGTGGCAG----AGGAGGCTATGG---AGGAGGCTA
L.calcarifer        TTTCAGACAAGGTGGTGAG--CGGGGTGGCAG----AGGAGGCTTTGG---AGGAGGTTA
E.fuscoguttatus     TTTCAGACAAGGTGGTGAC--CAGGGTGGCAG----AGGAGGCTTTGG---AGGAGGCTA
C.carpio            CTTCCGTGATGGTAGTGGTGATGAGGAGACAGGAAAAAGAGGCTTTGGTAGAGGAGGTTT
                     *** *    * * * *       **   **     * **** * ***   ****** *

D.labrax            CCGTGGAAAAGATGAGGAGGTCTTTTCTCAACG---GGAAGATAAAGA------AAATAA
L.japonicus         CCGTGGAAAAGATGAGGAGATATTTGCTCAACGTGGGGAAGATAAAGA------AAAGAA
T.orientalis        TCGTGGAAAAGATGAGGAGATCTTTACTCGAGG---GGAAGATAAAGA------TCCAGA
T.maccoyii          TCGTGGAAAAGATGAGGAGATCTTTACTCGAGG---GGAAGATAAAGA------TCCAGA
O.goramy            CCGTGGAAAAGATGAAGAGATCTTTGCTCAAGG---GGAAAATAAAGACCCAGGAAAGAA
L.calcarifer        CAGAGGAAAAGATGAACAGGTCTTTGCTCAAGG---GGAAGATAAAGATCC---AGGGAA
E.fuscoguttatus     CCGTGGGAAAGATGAGGAGGTCTTTGCTAAAGG---GGATGACAGAGATCC---AGAGAA
C.carpio            CAGAGGCCGGAATGAGGAAGTGTTTTC-CAAGGCCTCAACAGCAGACAAGCC--AGATCC
                      * **     ****  *  * *** *   * *     *    * * *

D.labrax            GGATGCAACCGATGGTGAAAGACCAAGGGTCACATATATTCCCCCAACCCTCCCTGAAGA
L.japonicus         GGATGCAAGTGATGGTGACAGACCGAAGGTCACGTATGTTCCCCCAACCCTCCCTGAAGA
T.orientalis        AAAGAAGGACGATAGTGACAGACCAAAGATCACATATGTTCCCCCAACCCTCCCTGAAGA
T.maccoyii          AAAGAAGGATGATAGTGACAGACCAAAGATCACATATGTTCCCCCAACCCTCCCTGAAGA
O.goramy            GGATGCGATCGATGGTGACAGACCGAAGGTAACATACGTTCCCCCAACACTCCCTGAAGA
L.calcarifer        TGATGCAAGTGAGGGTGACAGACCAAAGATCACATACATTCCACCAACCCTACCTGAAGA
E.fuscoguttatus     GGATGCAGGCGATGGTGACAAGCCAAAGGTCACGTATATTCCCCCGTCCCTTCCTGATGA
C.carpio            AGAAGCAGGTGAAAATGCAGGACCCAAGGTTGTCTATGTGCCGCCTCCCCCTCCAGAAGA
                      *       **   **     ** * * *    **  * ** **  * *  ** ** **

                                                                     Tpe-ddx4F1

                                                                     5′-GAYGABAT
D.labrax            TGAAGACTCCATTTTTGCCCACTATGAGACGGGCATCAACTTTGACAAGTATGATGACAT
L.japonicus         TGAAGACTCCATTTTTTCCCACTATGAGACGGGCATCAACTTTGACAAGTATGATGACAT
T.orientalis        TGAGGACTCCATTTTTTCCCACTATGAAACAGGGATCAACTTTGACAAGTATGATGACAT
T.maccoyii          TGAGGACTCCATTTTTTCCCACTATGAAACAGGGATCAACTTTGACAAGTATGATGACAT
O.goramy            CGAAGACTCCATTTTTGCACACTACAAGACAGGCATCAACTTTGACAAATATGATGACAT
L.calcarifer        CGAAGACGCCGTGTTTGCCCACTACGAGACAGGCATTAACTTTGATAAGTATGACGACAT
E.fuscoguttatus     CGAAGACTCCATTTTCGCCCACTACGAGTCAGGCATCAACTTCGACAAGTATGATGACAT
C.carpio            GGAGAGTTCCATATTTTCCCATTATGCCTCAGGCATTAATTTTGACAAATATGATGACAT
                     **     ** * **  * ** **     * ** ** ** ** ** ** ***** *****
```

```
                    MHTKGTVGAYGTBAGYGG-3’
D.labrax            CCTGGTGGATGTCAGTGGAGCCAATCCACCACAGGCTATAATGACTTTTGATGAGGCAGG
L.japonicus         CATGGTGGATGTCAGTGGTACCAACCCACCGCAGGCTATCATGACCTTTGATGAGGCAGC
T.orientalis        CATGGTGGATGTTAGTGGAACCAATCCACCACAAGCTGTCATGACTTTTGATGAGGCAGC
T.maccoyii          CATGGTGGATGTTAGTGGAACCAATCCACCACAAGCTGTCATGACTTTTGATGAGGCAGC
O.goramy            AATGGTGGATGTCAGCGGAACCAACCCACCACAGGCAATCCTGACCTTTGATGAGGCGGC
L.calcarifer        TATGGTTGATGTCAGTGGAACCAATCCACCAGAGGCACTCATGACTTTTGATGAGGCAAT
E.fuscoguttatus     CATGGTAGACGTCAGTGGAACCAACCCACCACAGGCCATCATGACTTTCGACGAGGCAGC
C.carpio            CCTTGTGGATGTGAGTGGCAGCAATCCTCCAAAGGCTATTATGACTTTTGATGAAGCAGG
                      * ** ** ** ** **   *** ** **  * **  *  **** ** ** ** **
```

Tpe-ddx4F2

```
                                                               5’-AAGCCBACYCCDGT
D.labrax            ATTGTGTGAGTCGCTGAGAAAAACAGTCAGCAAGTCTGGTTATGTGAAGCCGACCCCTGT
L.japonicus         ATTGTGCGAGTCCCTGAGAAAAAACGTCAGCAAATCTGGTTATGTAAAGCCGACCCCTGT
T.orientalis        ATTGTGCGAGTCCCTGAGAAAAAACGTCAGCAAGTCTGGTTATGTGAAGCCGACCCCTGT
T.maccoyii          ATTGTGCGAGTCCCTGAGAAAAAACGTCAGCAAGTCTGGTTATGTGAAGCCGACCCCTGT
O.goramy            GTTGTGCGAGACCCTGAGAAAAAACGTCAGCAAGTCTGGCTATGTGAAGCCAACCCCTGT
L.calcarifer        GTTGTGCGAATCCCTGAGAAAAAACGTCAGCAAGTCTGGTTACGTGAAGCCGACCCCTGT
E.fuscoguttatus     GTTGTGCGAGTCCCTGAGAAAAAACATCAAGAAGTCTGGTTACGTGAAGCCGACCCCTGT
C.carpio            ACTTTGTGAATCACTGAGAAAGAATGTAACGAAGTCTGGATATGTGAAGCCTACTCCTGT
                      * ** **  * ******** *   * *  ** ***** ** ** ***** ** *****
```

```
                    VCAGAARYAYGG-3’
D.labrax            GCAGAAGCATGGCATTCCAATCATCTCTGCTGGTAGAGATCTTATGGCCTGTGCCCAGAC
L.japonicus         TCAGAAGCACGGCATCCCAATCATCTCTGCTGGCAGAGATCTCATGGCCTGTGCCCAAAC
T.orientalis        GCAGAAGCACGGCATCCCAATCATCTCTGCTGGTAGAGATCTCATGGCCTGTGCCCAGAC
T.maccoyii          GCAGAAGCACGGCATCCCAATCATCTCTGCTGGTAGAGATCTCATGGCCTGTGCCCAGAC
O.goramy            GCAGAAGCACGGTATTCCAATCATTTCTGCCGGCAGAGATCTCATGGCCTGCGCCCAGAC
L.calcarifer        GCAGAAGCATGGCATCCCGATCATCTCTGCCGGCAGAGATCTCATGGCCTGTGCCCAGAC
E.fuscoguttatus     GCAGAAGTACGGCATCCCCATCATCTCTGCCGGCAGAGATCTCATGGCCTGTGCCCAGAC
C.carpio            CCAGAAACATGGAATCCCCATTATTTCTGCTGGAAGAGATCTAATGGCTTGTGCCCAGAC
                     *****  * ** ** ** ** ** ***** ** ******** ***** ** ***** **
```

```
D.labrax            TGGATCTGGTAAAACGGCTGCTTTCCTGCTCCCTATTCTGCAGCAGCTGATGGCAGACGG
L.japonicus         TGGATCTGGTAAAACGGCTGCATTCCTGCTCCCTATTCTGCAGCAACTGATGGCAGACGG
T.orientalis        TGGATCTGGTAAAACGGCTGCATTCCTGCTCCCTATTCTGCAGCAGCTGATGGCAGATGG
T.maccoyii          TGGATCTGGTAAAACGGCTGCATTCCTGCTCCCTATTCTGCAGCAGCTGATGGCAGATGG
O.goramy            TGGATCGGGCAAAACGGCTGCGTTCCTGCTCCCAATTCTGCAGCAGCTGATGGCAGACGG
L.calcarifer        TGGATCTGGTAAAACGGCTGCGTTCCTTCTCCCCATTCTGCAGCAGCTGATGGCGGACGG
E.fuscoguttatus     TGGATCTGGTAAAACGGCTGCGTTCCTGCTCCCCATTCTGCAGCAGCTGATGGCAGACGG
C.carpio            CGGGTCAGGAAAAACGGCGGCCTTCCTGCTGCCCATCCTACAGCGCTTTATGACTGATGG
                     ** ** ** ******** ** ***** ** ** ** ** ****   * *** * ** **
```

```
D.labrax            TGTGGCAGCCAGTTCCTTCAGTGAGCTGCAGGAGCCTGAAGCCGTCATCGTGGCTCCAAC
L.japonicus         TGTGGCAGCCAGTCGTTTCAGTGAGCTGCAGGAGCCTGAAGCCATCATTGTGGCCCCAAC
T.orientalis        TGTGGCAGCAAGTCGCTTCAGCGAGCTGCAGGAGCCTGAAGCAATCATTGTGGCCCCAAC
T.maccoyii          TGTGGCAGCAAGTCGCTTCAGCGAGCTGCAGGAGCCTGAAGCAATCATTGTGGCCCCAAC
O.goramy            AGTGGCAGCCAGTCGCTTCAGTGAGCTGCAGGAGCCTGAAGCTCTCATTGTGGCCCCGAC
L.calcarifer        TGTGGCAGCCAGTCGCTTCAGTGAGCTGCAGGAGCCTGAGGCCATCATTGTGGCCCCAAC
E.fuscoguttatus     TGTGGCAGCCAGTAGCTTCAGCGAGCTGCAGGAGCCTGAGACCATCATCGTGGCCCCGAC
C.carpio            TGTGGCAGCCAGCAAGTTCAGTGAGATACAGGAGCCTGAGGCCATAATCGTGGCTCCCAC
                     ******** **    ***** *** * ***********  *  * ** ***** ** **
```

```
D.labrax            TAGGGAGCTCATCAACCAGATTTACCTAGAGGCCAGGAAGTTCGCCTTTGGGACATGTGT
L.japonicus         TAGGGAGCTCATCAACCAGATTTACCTGGAAGCCAGGAAGTTTGCCTTTGGGACATGTGT
T.orientalis        CAGGGAGCTCATCAACCAGATTTACCTGGAGGCCAGGAAGTTTGCCTTTGGGACATGTGT
T.maccoyii          CAGGGAGCTCATCAACCAGATTTACCTGGAGGCCAGGAAGTTTGCCTTTGGGACATGTGT
O.goramy            CAGGGAGCTCATCAACCAGATTTACCTGGAGGCCAGAAAGTTTTCATTTGGGACGTGTGT
L.calcarifer        CAGGGAGCTCATCAATCAGATTTACCTAGAGGCCAGGAAGTTTGCTTTCGGGACGTGTGT
E.fuscoguttatus     CAGAGAACTCATCAACCAGATCTTCCTGGAGGCCAGGAAGTTTGCCTTTGGGACATGTGT
C.carpio            CAGAGAACTTATCAATCAGATCTATCTGGAGGCCAGGAAGTTTGCATACGGGACCTGTGT
                     ** ** ** ***** ***** *  ** ** ***** *****  * *  ***** *****
```

```
D.labrax             GCGTCCAGTAGTAGTCTATGGTGGAGTCAGCACTGGACATCAAATAAGAGAAATCTCAAG
L.japonicus          GTGTCCAGTAGTGGTTTATGGTGGAGCCAGCACTGGACACCAAATAAGGGAAATCTCAAG
T.orientalis         GCGTCCAGTGGTGGTTTATGGTGGAGTCAGCACTGGACACCAAATAAGAGAAATCGAAAG
T.maccoyii           GCGTCCAGTGGTGGTTTATGGTGGAGTCAGCACTGGACACCAAATAAGAGAAATCGAAAG
O.goramy             GCGTCCTGTAGTTGTTTATGGTGGAGTCAGCACTGCACACCAAATCAGAGAAATCTCAAG
L.calcarifer         GCGTCCGGTGGTTGTTTATGGTGGAGTCAGCACTGGATACCAAATAAGGGAAATATCAAG
E.fuscoguttatus      GCGTCCAGTCGTGGTGTATGGTGGAGTCAGCACTGGACACCAGATCAGAGAGATCTGCAG
C.carpio             ACGACCTGTTGTGGTTTATGGAGGTATAAATACCGGATATACTATCCGAGAGGTGTTAAA
                       * ** ** ** ** ***** **    *  ** * * *    **  * **  *    *
D.labrax             GGGATGCAATGTGCTGTGTGGAACACCAGGGAGACTGTTGGATGTGATTGGAAGAGGAAA
L.japonicus          GGGATGCAATGTTCTGTGTGGAACACCAGGGAGACTGTTGGATATGATTGGAAGAGGAAA
T.orientalis         GGGATGCAATGTAGTGTGTGGAACACCAGGGAGGCTATTGGATATGATTGGAAGAGGAAA
T.maccoyii           GGGATGCAATGTAGTGTGTGGAACACCAGGGAGGCTATTGGATATGATTGGAAGAGGAAA
O.goramy             GGGATGCAATGTGCTGTGTGGAACACCGGGGAGACTGTTGGATGTGATCGGAAGAGGAAA
L.calcarifer         GGGCTGCAATGTGCTGTGTGGAACACCAGGGAGACTGTTGGATATGATTGGAAGAGGAAA
E.fuscoguttatus      GGGCTGCAATGTGGTGTGTGGAACACCAGGGAGGCTGTTGGATGTGATCGGACGAGGAAA
C.carpio             GGGCTGCAATGTATTGTGTGCTACTCCTGGAAGATTGCATGACCTCATTGGTCGTGGAAA
                     *** ********  ******  ** ** ** **  *    **  * ** **  * *****

D.labrax             GGTTGGGTTGAGTAAGGTGCGGTACTTTGTGCTGGATGAGGCTGACCGGATGTTGGATAT
L.japonicus          GGTTGGGTTGAGTAAGTTGCGCTACTTTGTGCTGGACGAGGCTGACCGGATGTTGGATAT
T.orientalis         GGTTGGGTTGAGTAAGCTGCGGTACTTGGTGCTAGATGAGGCCGACCGGATGTTGGATAT
T.maccoyii           GGTTGGGTTGAGTAAGCTGCGGTACTTGGTGCTAGATGAGGCCGACCGGATGTTGGATAT
O.goramy             GGTTGGGTTGAGTAAGCTGAGGTACCTGGTGCTAGATGAGGCTGACAGGATGTTGGATAT
L.calcarifer         GGTTGGGCTGCAGAAGCTGCGGTACTTGGTGCTGGATGAGGCTGACCGTATGCTGGACAT
E.fuscoguttatus      GATTGGGTTGAGTAAGCTGCGCTACTTGGTGCTGGACGAGGCAGACCGGATGCTGGACAT
C.carpio             GATTGGCCTGAGTAAAGTGCGCTATCTAGTTCTGGATGAAGCAGACAGAATGCTGGACAT
                     * ****  **   **  ** * **  * ** ** ** ** ** *** * *** **** **

D.labrax             GG------GTTTTGAGCCTGACATGCGTCGCTTGGTGGGCTCCCCCGGAATGCCATCAAA
L.japonicus          GG------GTTTTGAGCCTGACATGCGCCGCTTGGTGGGCTCCCCTGGAATGCCGTCCAA
T.orientalis         GG------GATTTGAGCCTGACATGCGCCGCCTGGTGGGCTCACCTGGAATGCCATCCAA
T.maccoyii           GG------GATTTGAGCCTGACATGCGCCGCCTGGTGGGCTCACCTGGAATGCCATCCAA
O.goramy             GG------GTTTTGAGCCTGACATGCGACGCCTCGTGGGCTCTCCTGGAATGCCGTCCAA
L.calcarifer         GG------GCTTCGAGCCTGACATGCGGCGCCTGGTGGGCTCTCCTGGAATGCCATCCAA
E.fuscoguttatus      GG------GCTTTGAACCTGACATGCGCCGTCTGGTGGGCTCCCCTGGAATGCCACCCAA
C.carpio             GGACATGGGCTTCGAGCCAGATATGCGTAAGCTAGTGGGATCTCCGGGCATGCCTTCAAA
                     **      * ** ** ** ** *****     * ***** ** ** *****  * **

D.labrax             AGAGAACCGGCAGACGCTGATGTTCAGTGCCACCTACCCTGAGGACATTCAGAGGATGGC
L.japonicus          AGAGAACCGTCAGACTCTGATGTTCAGTGCCACCTACCCTGAGGACATTCAGAGGATGGC
T.orientalis         AGAGAACCGTCAGACTCTGATGTTCAGTGCCACATACCCTGAAGACATCCAGAGGATGGC
T.maccoyii           AGAGAACCGTCAGACTCTGATGTTCAGTGCCACATACCCTGAAGACATCCAGAGGATGGC
O.goramy             AGAGAACCGTCAGACTCTGATGTTCAGTGCCACCTACCCTGAGGACATCCAGAGGATGGC
L.calcarifer         GGAGGACCGTCAGACTCTGATGTTCAGTGCCACCTATCCTGAGGACATCCAAAGGATGGC
E.fuscoguttatus      AGAGAGCCGCCAGACTCTGATGTTCAGCGCCACCTACCCCGAGGACATCCAGAGGATGGC
C.carpio             AGAGGATCGACAAACCCTCATGTTCAGTGCCACCTACCCTGAAGATATTCAAAGGATGGC
                      ***   ** ** ** ** ******** ***** ** ** ** ** ** ** ********

D.labrax             AGCAGACTTTCTCAAGACCGACTATTTGTTCTTGGCTGTGGGTGTGGTTGGCGGAGCCTG
L.japonicus          AGCTGACTTCCTCAAGACGGACTATTTATTCTTGGCTGTGGGTGTGGTGGGCGGAGCCTG
T.orientalis         GGCTGACTTCCTCAAGACAGACTATTTGTTCCTGGCTGTGGGTGTGGTGGGTGGAGCCTG
T.maccoyii           TGCTGACTTCCTCAAGACAGACTATTTGTTCCTGGCTGTGGGTGTGGTGGGTGGAGCCTG
O.goramy             GGCTGACTTCCTGAAGACAGACTATTTGTTCTTGGCTGTGGGTGTGGTGGGTGGAGCCTG
L.calcarifer         GGCAGACTTCCTCAAGACTGACTATTTGTTCCTGGCTGTGGGTGTGGTGGGAGGAGCCTG
E.fuscoguttatus      CGCCGACTTCCTCAAGACTGACTATTTGTTCTTGGCTGTGGGTATTGTGGGCGGAGCCTG
C.carpio             TGCAGATTTTTTGAAAGTGGACTACATTTTCCTTGCTGTTGGTGTGGTTGGTGGAGCATG
                      ** ** **  * **    *****  * *** * ***** *** * ** ** ***** **

D.labrax             CAGTGATGTGGAGCAGACATTTGTCCAGGTCACCAAGTTCTCCAAAAGGGAGCAACTCCT
L.japonicus          CAGTGACGTGGAGCAGACATTTGTCCAAGTCACTAAGTTCTCCAAGAGGGAGCAGCTCCT
T.orientalis         CAGTGATGTGGAGCAGACATTTATCCAAGTCACAAAGTTCTCCAAGAGAGAGCAGCTCCT
T.maccoyii           CAGTGATGTGGAGCAGACATTTATCCAAGTCACAAAGTTCTCCAAGAGAGAGCAGCTCCT
O.goramy             CAGTGATGTGGAGCAGACATTTGTCCAAGTGACCAAATTCTCCAAGAGGGAGCAGCTCCT
L.calcarifer         CAGCGATGTGGAGCAGACATTTATTGAAGTTACCAAATTCTCCAAGAGGGAGCAGCTACT
E.fuscoguttatus      CAGTGACGTGGAGCAGTCATTTGTGCAAGTCACCAAGTTCTCCAAGAGGGAGCAGCTCCT
C.carpio             CAGTGATGTGGAGCAAACTATCGTTCAGGTGACCAG---TACCAAGAGAGACCAGCTGCT
                     *** ** ********  *  *  *  * ** ** *      **** ** ** ** ** **
```

```
D.labrax            TGACCTCCTCAAGACAACTGGAATGGAGCGCACTATGGTGTTTGTGGAGACCAAGAGACA
L.japonicus         TGACCTCCTCAAGTCAACTGGAATGGAGCGCACCATGGTGTTTGTAGAGACCAAGAGACA
T.orientalis        TGACCTCCTGAAGACCACTGGAACGGAGCGCACCATGGTGTTTGTAGAGACCAAACGACA
T.maccoyii          TGACCTCCTGAAGACCACTGGAACGGAGCGCACCATGGTGTTTGTGGAGACCAAACGACA
O.goramy            GGATCTCCTGAAGACCACAGGAACGGAGCGCACCATGGTGTTTGTGGAGACCAAGAGACA
L.calcarifer        TGACCTCCTGAAGACCACAGGAACGGAGCGCACCATGGTGTTTGTGGAAACCAAGAGACA
E.fuscoguttatus     CGACCTCCTCAAGACCACTGGAACGGAGCGCACCATGGTGTTTGTGGAGACCAAGAGACA
C.carpio            TGAACTGCTCAGATCTACAGGGAATGAACGCACAATGGTCTTTGTTGAAACCAAAAGAAG
                     ** ** ** *   * ** ** *  ** ***** ***** ***** ** *****  **

D.labrax            AGCTGATTTCATTGCCACTTACTTGTGCCAGGAAAAGGTTCCTACCACCAGCATTCATGG
L.japonicus         AGCTGATTTTATTGCCACTTACTTGTGCCAGGAAAAGGTTCCAACTACAAGCATTCATGG
T.orientalis        AGCTGATTTTATTGCCACGTTCTTGTGCCAAGAGAAAGTTCCAACTACCAGCATTCACGG
T.maccoyii          AGCTGATTTTATTGCCACGTTCTTGTGCCAAGAGAAAGTTCCAACTACCAGCATTCACGG
O.goramy            AGCTGATTTTATTGCCACATTCTTGTGCCAGGAGAAGGTTCCCACAACCAGCATTCATGG
L.calcarifer        GGCTGATTTTATTGCCACTTTCCTGTGCCAGGAGAAGGTTCCAACCACCAGCATTCATGG
E.fuscoguttatus     AGCTGATTTCATCGCCACTTATTTGTGCCAGGAGAAGGTTCCAACTACGAGCATCCACGG
C.carpio            TGCTGATTTCATTGCAACATTTCTCTGTCAAGAGAAGATGTCTACTACAAGCATCCATGG
                     ******** ** ** ** *   * ** ** ** **  *  * ** ***** ** **

D.labrax            TGACCGAGAGCAGCGGGAGCGAGAGCAGGCTCTGGCAGACTTCCGCTCTGGCAAATGTCC
L.japonicus         TGACCGTGAGCAGCGGGAGCGAGAGCAGGCTCTGGCCGACTTCCGCTCTGGCAAATGTCC
T.orientalis        TGACCGAGAGCAGCGGGAGCGAGAGCAGGCTCTGGCAGACTTCCGCTCTGGTAAATGTCC
T.maccoyii          TGACCGAGAGCAGCGGGAGCGAGAGCAGGCTCTGGCAGACTTCCGCTCTGGTAAATGTCC
O.goramy            TGACCGTGAGCAGCGGGAGCGCGAGCAGGCTCTGGCAGACTTCCGCTCTGGTAAATGTCC
L.calcarifer        TGACCGCGAGCAGCGTGAGCGAGAGCAGGCCCTGGCAGACTTCCGCTCCGGCAAGTGTCC
E.fuscoguttatus     GGACCGTGAGCAGCGGGAGAGAGAGCAGGCTCTGGCTGACTTCCGCTCCGGCAAATGTCC
C.carpio            TGATCGGGAGCAACGAGAGCGAGAGAAGGCTCTCAGTGATTTTCGCACAGGCCAGTGTCC
                     ** ** ***** ** *** * *** **** **    ** ** *** * **  * *****

D.labrax            AGTCCTAGTGGCAACCTCTGTGGCTGCCCGTGGTCTGGACATTCCAGATGTACAGCATGT
L.japonicus         AGTCCTGGTGGCAACCTCGGTAGCTGCCCGTGGTCTGGATATTCCAGATGTACAGCATGT
T.orientalis        AGTCCTAGTAGCAACCTCTGTAGCTGCCCGCGGTCTGGATATTCCAGATGTACAGCATGT
T.maccoyii          AGTCCTAGTAGCAACCTCTGTAGCTGCCCGCGGTCTGGATATTCCAGATGTACAGCATGT
O.goramy            TGTCCTGGTGGCGACGTCTGTAGCTGCCCGCGGTCTGGATATTCCTGATGTACAACATGT
L.calcarifer        GGTCCTGGTGGCCACCTCTGTCGCCGCCCGTGGCCTGGATATTCCAGATGTGCAGCATGT
E.fuscoguttatus     CGTCCTGGTGGCAACTTCTGTCGCTGCCCGCGGTCTGGATATTCCCGATGTACAGCACGT
C.carpio            AGTGCTGGTGGCCACGTCAGTCGCTGCCAGAGGCCTGGACATTGAGCAGGTCCAGCACGT
                     ** ** ** ** ** ** ** ** *** * ** ***** ***    * ** ** ** **

                                                                      Tpe-ddx4R2
                                                                3′-TYGGVMGMACHGG
D.labrax            GGTGAACTTTGACCTCCCCAACAACATTGACGAATATGTCCACCGTATTGGGAGAACTGG
L.japonicus         GGTGAACTTTGACCTCCCCAACAACATTGACGAATATGTCCACCGTATTGGAAGAACTGG
T.orientalis        GGTTAACTTTGACCTCCCCAACAACATTGATGAATATGTCCACCGTATTGGGAGGACTGG
T.maccoyii          GGTTAACTTTGACCTCCCCAACAACATTGATGAATATGTCCACCGTATTGGGAGGACTGG
O.goramy            GGTGAACTTTGACCTCCCCAGCAACATCGACGAATACGTGCACCGCATTGGGAGAACTGG
L.calcarifer        GGTGAACTTTGACCTCCCCAACAACATTGATGAATATGTCCACCGTATTGGGAGAACCGG
E.fuscoguttatus     GGTGAACTTTGACCTCCCCAACAACATCGACGAGTACGTCCACCGTATTGGGAGAACTGG
C.carpio            TGTGAATTTCGACCTGCCCAGCAGCATCGACGAATACGTCCATCGCATCGGAAGAACCGG
                     ** ** ** ***** **** ** *** ** ** ** ** ** ** ** ** ** ** **

                    HCGMTGYGGDAA-5′
D.labrax            CCGCTGTGGTAACACTGGAAGGGCGGTGTCTTTCTTTGACCCAGATTCTGATGACCAATT
L.japonicus         CCGCTGTGGAAACACTGGGAGGGCTGTCTCTTTCTATGACCCAGATGCTGATGGCCAATT
T.orientalis        CCGCTGCGGTAACACAGGGAGGGCAGTGTCTTTCTATGACCCTGATGCTGATGGCCAACT
T.maccoyii          CCGCTGCGGTAACACAGGGAGGGCAGTGTCTTTCTATGACCCTGATGCTGATGGCCAACT
O.goramy            CCGCTGTGGGAACACTGGGAGGGCGGTGTCTTTCTATGACCCAGAAGCTGATGGCCATTT
L.calcarifer        CCGCTGCGGTAACGTCGGGAGGGCGGTGTCTTTCTACGACCCAGACGCTGACGTCCAGCT
E.fuscoguttatus     CCGCTGTGGGAACACTGGGAGGGCGGTGTCTTTCTACGACCCAGATAATGATAACCAGTT
C.carpio            ACGCTGTGGGAACATCGGGCGGGCCGTGTCCTTTTTTAACCCAGAGTCTGACACTCCATT
                     ***** ** ***   **  **** ** ** ** *   **** **   ***    *   *
```

Tpe-ddx4R1

3′-CCYCARCAGGARGTKCCBWMVTGG-5

```
D.labrax           GGCTCGCTCCCTAGTCACAATCCTGTCCAAGGCTCAGCAGGAAGTGCCTTCATGGTTGGA
L.japonicus        GGCTCGCTCCCTGGTCACAGTCCTGTCCAAGGCCCAGCAGGAAGTGCCCTCATGGTTAGA
T.orientalis       GGCTCGCTCCTTGGTCACAGTCCTGTCCAAGGCCCAGCAGGAAGTGCCTTCATGGTTAGA
T.maccoyii         GGCTCGCTCCTTGGTCACAGTCCTGTCCAAGGCCCAGCAGGAAGTGCCTTCATGGTTAGA
O.goramy           GGCTCGTTCCCTGGTCGGTGTCCTGTCCAAGGCCCAGCAGGAGGTGCCCTCATGGTTAGA
L.calcarifer       GGCTCGATCCTTGGTCACAGTCCTCTCCAAGGCTCAGCAGGTAGTGCCCTCATGGTTAGA
E.fuscoguttatus    GGCCCGCTCTCTGGTCACGATCCTGTCCAAGGCTCAACAGGAAGTGCCCGCGTGGTTAGA
C.carpio           AGCTCGCTCTCTGGTCAAAGTTCTGTCTGGGGCCCAACAGGAAGTTCCCAAATGGCTGGA
                    ** ** **  * ***    * ** **   *** ** ****  ** **    *** * **

D.labrax           GGATTCTGCATTCAGTGGCCC---TGGTAGCATGGGCGTCG---CCCCCAGAAAGACATT
L.japonicus        GGAGTTTGCGTTCAGCGGCCC---TGCAACCACAGGCTTCAATCCTCCCAGGAAGAACTT
T.orientalis       AGAGTCTGCGTTCAGCGGACC---TGCTACCACTGGCTTTAACCCACCTAGGAAGAACTT
T.maccoyii         AGAGTCTGCGTTCAGCGGACC---TGCTACCACTGGCTTTAACCCACCTAGGAAGAACTT
O.goramy           GGAGGCTGCGTTCAGTGGCCC---CAGTTCCACAGGCTTCAACCCACCCAGGAAGAACTT
L.calcarifer       GGAGCACGCATTCTCTGGCCT---CAGT---ACAGGCTTCAACCCCCCCAGGAAGAACTT
E.fuscoguttatus    GGAGTCGGCGTTCAGCGGCGGGGGCGGAGGCTCAGGCTTCAACGCCCCCAGGAAAAGCTT
C.carpio           GGAAATTGCTTTCAGTGCTCA---TGGAACAACAGGTTTTAACCCTCGTGGAAAAGTGTT
                    **    ** ***   *                 **  *     * *   * **    **

D.labrax           TGCCTCCTCAGACTCCAGAAAGGGCCCACAGGGAGGATCTGTCCAAGAC-----GACAGC
L.japonicus        TGCCTCCTCAGACTCCAGGAAGGG---------AGGATCTTTCCAAGAC-----AATGGT
T.orientalis       TGCCTCCACAGACTCCAGGAAGAG---------AGGATCTTTCCAAGAC-----AACAGT
T.maccoyii         TGCCTCCACAGACTCCAGGAAGAG---------AGGATCTTTCCAAGAC-----AACAGT
O.goramy           TGCCTCCACAGACACCAGACAGAG---------AGGATTGTTGCAAGAC-----ACCAGT
L.calcarifer       TGCCTCCACAGACTCCAGGAAGGG---------AGGATCCTTTCAGGAC-----AACATG
E.fuscoguttatus    TGCCTCCACAGACTCCAGGAAGGG---------AGGATCTTCCCAGGAC-----AACAGT
C.carpio           TGCGTCTACAGACACCCGGAAGGG---------AGGGTCCTT-CAAGACGGATGAGCCGC
                   *** **  ***** ** *  ** *         *** *     ** ***

D.labrax           GTGCAGAGCCAGTCGGCTGCTCAGACTGCAC---ATGATGAAGAAGAATGGGATTAG
L.japonicus        ATGGAGAGCTTGCCGGCTGCTCAGGCTGCAG---CTGATGATGAAGAATGGGAGTAG
T.orientalis       GTGAAGAGCCAGCCGGCTGTTCAGACTGCAGCGGATGATGATGAGGAATGGGAATAG
T.maccoyii         GTGAAGAGCCAGCCGGCTGTTCAGACTGCAGCTGATGATGATGAGGAATGGGAATAG
O.goramy           GTGATGAGCCAGCCGGCTGCTCAGCCTGCAG---CTGATGAAGAAGAGTGGGAGTAG
L.calcarifer       ATGAAGAGCCAGCCGGCTGCTCTGGCTGCAG---CTGACGAGGAAGAATGGGAATAG
E.fuscoguttatus    GTGAAGAGTCCTCCAGCTGCTCAGTCAGCTA---ACGATGATGAAGAGTGGGAATAG
C.carpio           CTCAAGCATCCGCCCCAAGCCCAG-CAGCTGCTGCAGATGATGAAGAATGGGAATAA
                    *   *       *    *  * * * **       ** ** ** ** ***** **
```

S2A. Multiple alignment of amino acid of the known piscine Dnd protein homologs and location of degenerate primer design. *M. anguillicaudatus*, BAJ19134.1; *D. rerio*, NP_997960.1; *C. gibelio*, AKL72310.1; *G. rarus*, AIF74583.1; *O. mykiss*, NP_001118133.1; *S. salar*, NP_001266060.1; *O. latipes*, NP_001157988.1; *O. mossambicus*, ALM89050.1; *P. olivaceus*, AKD44207.1

```
                                                                       Tpe-dndF1
M.anguillicaudatus      -------------------MEGADLLQLFNPQALKALKEWMQKSSTTLTQVNGQRKYGGP 41
D.rerio                 -------------MVGDMDAQQQELQQILNPQKLKSLQEWMQRNSITLTQVNGQRKYGGP 47
C.gibelio               -------------------MEGQQLQQLLNPQRLKSLQEWMQKSSITLTQVNGQRKYGGP 41
G.rarus                 -------------------MDG-QQQQVLNPQRLKSLKEWVQKSSITLTQVNGQRKYGGP 40
O.mykiss                --------------------MEERSSQVLNPERLKALEMWLQETDVKLTQVNGQRKYGGP 40
S.salar                 --------------------MEERSSQVLNPERLKALEMWLQETDVKLTQVNGQRKYGGP 40
O.latipes               ---------------------MDNQSKVVNLERVQALQAWVKSTNTKLTQVNGQRKYGGP 39
O.mossambicus           --------------------MENTQSQVLNLERVQALEIWLKATNTKLTQVNGQRKYGGP 40
P.olivaceus             MQPRGDREASQNPAMEEE-VMEAAQSQVLNFEPVPALETWLKTTNTQLIQVNGQRKYGGP 59
                                                  ::.* : : :*: *:: ..  * ***********

                                                                Tpe-dndR1
M.anguillicaudatus      PPGWHGPAPGPGCEVFISHIPRNVFEDTLIPLFQRVGTLYEFRLMMNFSGQNRGFAYAKY 101
D.rerio                 PPGWQGPAPGSGCEVFISQIPNDVYEDRLIPLFQSIGTIYEFRLMMNFSGQTRGFAYAKY 107
C.gibelio               PPGWRGPVPGPGCEVFISQIPRDVYEDRLIPLFQSIGTIYEFRLMMNFSGQNRGFAYAKY 101
G.rarus                 PPGWQGPAPGPGCEVFISQIPRDVYEDRLIPLFQSVGTLYEFRLMMNFSGQNRGFAYAKY 100
O.mykiss                PDDWLGAPPGPGCEVFISQIPRDVFEDQLIPLFRAVGPLWEFRLMMNFSGQNRGFAYAKY 100
S.salar                 PDDWLGAPPGPGCEVFISQIPRDVFEDQLIPLFRAVGPLWEFRLMMNFSGQNRGFAYAKY 100
O.latipes               PDVWDGPPPGARCEVFISQIPRDVYEDLLIPLFSSVGALWEFRLMMNFSGQNRGFAYAKY 99
O.mossambicus           PEVWEGPTPGPRCEVFISQIPRDAYEDILIPLFSSIGPLWEFRLMMNFSGQNRGFAYAKY 100
P.olivaceus             PEVWNGPIPGAHCEVFISQIPRDAYEDLLIPLFSSVGPLWEFRLMMNFSGQNRGFAYAKY 119
                        *  * *  **  ******:**.:.:** *****  :* ::***********.********

M.anguillicaudatus      GDQVTAITAVKTLNQHRIADGVRLMVRRSMEKRQLWIE---DLPPDVSQDELLMMLRQIS 158
D.rerio                 GDPLTASAAVTTLHQYRLPEGGCLTVRRSTEKRQLRLG---DLPVSMNESKLLMVLQMLS 164
C.gibelio               GDPLTASTAVLTLHHYRLPEGGCLTVRNSTEKRQLRLG---DLPTSINQVELLRMLRMMS 158
G.rarus                 GDPFTASTAVMTLHQYPLQEGVCLTVRRSTEKRQLRLG---DLPTNVNQTEMLMVLQMMS 157
O.mykiss                DSPASAAAAIRSLHGRALESGARLSVRRSTEKRQLCLG---ELPTSTRREQLLQVLLDFS 157
S.salar                 DSPASAAAAIRSLHGRALESGARLGVRRSTEKRQLCLG---ELPTSTRREQLLQVLLDFS 157
O.latipes               GTAAIANDAIHLLHGYPLGPGARLSVRCSIEKRQLCIQ---NLPASTRQEELLQVLRLLS 156
O.mossambicus           GSAAIAVEAIRQLHGHMVEPGYRISVRRSTEKRHLCIGGIGGLPASTRQEGILQVLRMLV 160
P.olivaceus             SSSGLAAEAICLLDGYMVEQGSCIKVRLSIEKRHLCIE---DLPATTRQEELLEVLRVLT 176
                        .    *  *:  *.   :  *  : ** * ***:* :     **    .  :* :*  :

M.anguillicaudatus      EGVEDVTLKFSGPKGGDVAAVVSYFTHIAASMANKVLVEAFKKQYGMSVTVRWMTFTAKS 218
D.rerio                 DGVEDVLLKPPGPKGKEVVALVNYTSHYAASMAKKVLVEAFRNRYGISITVRWTSFS-KS 223
C.gibelio               EGVEEVLLKMAGPKGREFVALVNYSSHYAASMAKKVLVQAFRKQYGISITVKWMSFS-KS 217
G.rarus                 EGVENVLLKLAGPKGREVVALVNYSSHYAASMAKKVLVEAFKKQCGISITVSWTSFS-KS 216
O.mykiss                EGVEGVSLR-AGPREQGLSAVVVYASHHAASMAKKVLIEAFKKRFGLAITLKWQSSSRPK 216
S.salar                 EGVEGVSLR-AGPGEQGMSAVVVYASHHAASMAKKVLIEAFKKRFGLAITLKWQSSSRPK 216
O.latipes               AGVESVALK-AGPGIEGVSAVVAFSSHHAASMAKKALGEEFKKQFCLDISIKWLSAEKPN 215
O.mossambicus           EGVERVSLK-AGPGIEGVSATVAFSSHHAASMAKKVLVEAFKKQFTMCVSVKWQPTEKPN 219
P.olivaceus             EGVQKVSLT-AGPGIEGVSAIVAFLSHYTASMAKKMLVEVFKKQFALNISIVWHPDESLS 235
                         **: * *   **    . * * : :* :****:* * : *:::  : ::: *      .

M.anguillicaudatus      KHVEKTKQDNGPPAPPSLKPLSKPF---SHLHTQPS-----HHIIPTHTTPQPFTRAVGA 270
D.rerio                 KRVEDTPQEDSCVTPLVLKPLSKPS---------LL-----HYDVPAHQSLLPLFRAVGG 269
C.gibelio               KHVDETGED-DRFTPPVLNPLPKPSITPPHLHPQPL-----LHDVPPHPLIQPFFRAAGG 271
G.rarus                 KRVKEMGQEDDFFTTPGLKPLTKPSLSPPCLYPQLS-----HHDVPAHPTLQPFFRAVGG 271
O.mykiss                HEEPPRPS----KTPPS----PKPP--HCSLLDSPRPPL-----RLAQRQLPAFSRAVRA 261
S.salar                 HEEPPRPS----KTPPSSP--PKPP--RCSLLDSPRPPL-----HLAQRQLPAFSRAVRA 263
O.latipes               PDKPPP-Q----RAPKGLLPSPLKH--L----GQTSPRLPPRL--ASPAVPTAFCKAVGG 262
O.mossambicus           PDEPRCPQ----KRAKSLLPSHLGP--L--HHSSPQPSGPPSFLTLPASIPAGFCRAVGG 271
P.olivaceus             PNKPPKS----------PLASPLKP--PCHILKSPQPSVPPPHLAHHSPTPPSFCRAVGG 283
                                                                             : :*. .
```

```
M.anguillicaudatus      PVTQPRNEP----------------------------------------ISQSRDAASQL 290
D.rerio                 PTTSEQRDEMIPQPTIMSRNELIPQSSIRQRDEMVPQLPIRPRDGMAPQSPISLDAVSHL 329
C.gibelio               PAGLRDETM--------------------------------------PRAPVNQDAVSQL 293
G.rarus                 PTAPPRDEMMAP-----------------------------------NPNPVNRDTVSQL 296
O.mykiss                PPPMVHAA-----PESLREV----------------------------TVVPPVDAAALL 288
S.salar                 PSPMVHAA-----PESPRGA----------------------------TMVPPVDAAALL 290
O.latipes               PPTQHDTHVKGT--SPPPRG----------------------------QVMFSVSPVLLL 292
O.mossambicus           PTAPQLSHPTCSFPDSSTQG----------------------------HLVFAASPVMFL 303
P.olivaceus             PTAFSHPHCPHHFTFSSSQG----------------------------DRVPVESPVMLL 315
                        *                                                     . .  *

M.anguillicaudatus      KWLCEVNKLGTPRYEARYNHIGPDGFL-YFTYKVMIPALSFPLYGSVQILPGTSARAMKG 349
D.rerio                 QWMCEVNRLGSPQYEVHFHHAAPDGFL-YFAFKVLIPGLPLPLYGFVQILPGTSARAMKS 388
C.gibelio               QWICELHRLGTPQYEVRFHRTSSDGFL-YFNFKVLVPGLLFPLNGVIQILPGTSIQAMKA 352
G.rarus                 QSMCEANRLGAPQYEVRYHHTGPDGLSSTFAFKVLIPGLLLPLFGAVQILPGTSAQAMKG 356
O.mykiss                QGVCEVYGQGKPLYDLQYRHMGPDGFL-CFSYRVYVPGLATPFTGMVQTLPGPTPGAIQE 347
S.salar                 QGVCEVYGQGKPLYDLQYRHMGPDGFL-CFSYRVYVPGLATPFTGMVQTLPGPTPGAIQE 349
O.latipes               RKLSEASGWGDPHYEMLFSHAGPDGFL-YFTYKVHVPGAPTTFRGFVMILPGHCTSTMLE 351
O.mossambicus           SKLCETAGVGQPFYEVQYSHAGPDGFL-YFSYKVQIRGITAAFKGLVMILPGPNVSTMLE 362
P.olivaceus             SKVCEVTGVGQPRYEMSYSHTGRNGFL-YFDYTVCIPRITTPFKGLVMTLPGPTASTVQE 374
                          :.*    * * *:  : : . :*:   * : * :      : * :  ***    ::

M.anguillicaudatus      EVHRATAEQVIQTIYTAARWQPF---------   372
D.rerio                 EVYRAAAEQVIQTLCRVSNLRPF---------   411
C.gibelio               EVYRAAAEQVIQTMCKVSNLRPF---------   375
G.rarus                 EVCRAAAEQVIQTMCKGLKFATFLKFELFLLM   388
O.mykiss                EARRATAQQVLSALYRA---------------   364
S.salar                 EARRATAQQVLSALYRA---------------   366
O.latipes               EARRAAAQQVLQKLCSSGLSA-----------   372
O.mossambicus           EAQKAVAQQVLQKMYNTGLTH-----------   383
P.olivaceus             EAGQAAAQQVLKRIYNNQ--------------   392
                        *. :*.*:**:. :
```

S2B. Nucleotide alignment of the known piscine deadend homologs and degenerate oligonucleotide primer design for cloning of *dnd1*. *M. anguillicaudatus*, BAJ19134.1; *D. rerio*, NP_997960.1; *C. gibelio*, AKL72310.1; *G. rarus*, AIF74583.1; *O. mykiss*, NP_001118133.1; *S. salar*, NP_001266060.1; *O. latipes*, NP_001157988.1; *O. mossambicus*, ALM89050.1; *P. olivaceus*, AKD44207.1

```
O.mykiss                ---------------------------------------------------------ATG
S.salar                 ---------------------------------------------------------ATG
C.gibelio               ------------------------------------------------------ATGGAG
D.rerio                 ------------------------------------ATGGTCGGAGACATGGATGCCCAG
G.rarus                 ------------------------------------------------------ATGGAT
M.anguillicaudatus      ------------------------------------------------------ATGGAA
O.latipes               ------------------------------------------------------------
P.olivaceus             ATGCAGCCGCGGGGCGACAGAGAGGCGTCACAGAACCCAGCGATGGAGGAGGAGGTGATG
O.mossambicus           ---------------------------------------------------------ATG


O.mykiss                GAGGAGCGTTCAAGTCAGGTGTTGAACCCGGAGCGACTGAAGGCGCTGGAGATGTGGCTG
S.salar                 GAGGAGCGTTCAAGTCAGGTGTTGAACCCGGAGCGACTGAAGGCGCTGGAGATGTGGCTG
C.gibelio               GGACAGCAGCTACAGCAGCTTTTGAACCCGCAGAGACTGAAGTCACTTCAAGAATGGATG
D.rerio                 CAGCAGGAGCTTCAGCAGATTCTGAACCCGCAGAAACTCAAGTCTCTGCAGGAATGGATG
G.rarus                 GGACAGCAGC---AGCAGGTTTTGAACCCGCAGAGACTGAAGTCACTAAAAGAATGGGTG
M.anguillicaudatus      GGAGCAGATCTGCTACAGCTTTTCAACCCGCAGGCACTGAAGGCACTGAAGGAGTGGATG
O.latipes               ATGGACAATCAGAGCAAGGTGGTGAACTTGGAGCGGGTCCAGGCACTTCAGGCCTGGGTC
P.olivaceus             GAGGCCGCGCAGAGCCAGGTCCTGAACTTTGAGCCGGTGCCAGCGCTGGAAACCTGGCTG
O.mossambicus           GAAAACACGCAAAGCCAGGTGCTGAACCTTGAACGGGTGCAGGCCCTGGAAATCTGGTTG
                                        ** *  * ***    *     *     * **  *    *** *

                                                          Tpe-dndF1
                                                    5'-AAYGGNCARMGNAARTAYGG-3'
O.mykiss                CAGGAGACTGACGTCAAACTGACCCAAGTCAATGGCCAGAGAAAATATGGAGGTCCACCT
S.salar                 CAGGAGACTGACGTCAAACTGACCCAGGTCAATGGCCAGAGGAAATATGGAGGTCCACCT
C.gibelio               CAGAAAAGCTCAATCACTTTAACACAGGTCAATGGGCAGAGGAAGTATGGTGGTCCTCCT
D.rerio                 CAGAGGAACTCCATCACTTTAACCCAAGTCAATGGGCAGAGGAAATATGGTGGTCCTCCT
G.rarus                 CAGAAAAGCTCCATCACTTTAACACAGGTCAACGGGCAGAGGAAATATGGTGGTCCTCCT
M.anguillicaudatus      CAAAAATCCTCCACCACCCTAACGCAGGTCAACGGTCAGAGGAAATATGGTGGTCCTCCT
O.latipes               AAGTCCACCAATACCAAACTGACCCAGGTTAACGGCCAGAGGAAGTATGGAGGGCCACCT
P.olivaceus             AAAACAACCAATACACAACTGATTCAAGTAAATGGCCAGAGGAAGTATGGAGGACCACCT
O.mossambicus           AAAGCAACCAACACAAAGCTGACTCAAGTTAATGGCCAGAGGAAATATGGAGGACCACCT
                         *                 * *  ** ** ** ** ***** ** ***** ** ** ***

O.mykiss                GATGATTGGCTTGGCGCCCCCCCTGGGCCGGGCTGTGAGGTGTTCATCAGCCAGATCCCG
S.salar                 GATGACTGGCTTGGCGCCCCCCCTGGGCCGGGCTGTGAGGTGTTCATCAGCCAGATCCCG
C.gibelio               CCGGGCTGGCGAGGTCCTGTTCCCGGGCCGGGCTGTGAGGTTTTCATCAGTCAGATCCCG
D.rerio                 CCAGGTTGGCAGGGTCCTGCTCCTGGTTCGGGCTGTGAGGTTTTCATCAGTCAGATCCCG
G.rarus                 CCGGGCTGGCAAGGTCCTGCTCCCGGCCCAGGCTGTGAGGTTTTCATCAGTCAGATCCCA
M.anguillicaudatus      CCGGGCTGGCACGGTCCTGCTCCAGGGCCAGGTTGCGAGGTCTTTATCAGTCATATTCCA
O.latipes               GATGTGTGGGACGGTCCCCCACCTGGGGCGCGCTGTGAGGTCTTTATCAGCCAGATCCCA
P.olivaceus             GAGGTGTGGAACGGCCCCATCCCAGGAGCCCACTGCGAGGTCTTCATCAGCCAGATCCCA
O.mossambicus           GAGGTGTGGGAAGGTCCCACACCGGGACCGCGCTGTGAAGTCTTCATCAGCCAGATCCCA
                           *  ***   **  *    ** **  *    ** ** ** ** ***** ** ** **

O.mykiss                CGGGATGTCTTTGAGGACCAGCTGATTCCGCTGTTCCGTGCGGTGGCCCTCTCTGGGAG
S.salar                 CGGGATGTCTTTGAGGACCAGCTGATTCCGCTGTTCCGTGCCGTGGCCCTCTCTGGGAG
C.gibelio               CGCGACGTCTACGAGGACCGCCTGATCCCTCTTTTCCAGAGCATTGGCACTATTTACGAG
D.rerio                 AACGACGTGTACGAGGACCGCCTGATCCCTCTCTTCCAGAGCATCGGCACCATTTACGAG
G.rarus                 CGTGATGTCTACGAGGACCGATTGATTCCTCTCTTCCAGAGCGTCGGTACACTTTATGAG
M.anguillicaudatus      CGTAACGTCTTTGAGGACACCCTAATCCCGCTCTTCCAGAGAGTCGGCACTCTATACGAG
O.latipes               CGGGATGTCTACGAGGACCTGCTCATCCCCCTCTTCAGCTCGGTGGGGGCACTTTGGGAA
P.olivaceus             CGGGACGCCTACGAGGACCTGCTGATCCCCCTGTTCAGCTCTGTGGGGCCTCTCTGGGAG
O.mossambicus           CGGGACGCGTACGAGGACATCCTTATTCCCCTGTTCAGCTCCATTGGCCACTCTGGGAG
                            * *  *  ******    * ** ** ** ***       * **  *  * *  **
```

Tpe-dndR1

```
                              3′-ATGATGAAYTTYWSNGGNCA-5′
O.mykiss                TTCCGCCTCATGATGAACTTCAGCGGACAGAACCGTGGCTTTGCCTACGCCAAGTACGAC
S.salar                 TTCCGCCTCATGATGAACTTCAGCGGACAGAACCGTGGCTTTGCCTACGCCAAGTACGAC
C.gibelio               TTTCGTCTCATGATGAACTTCAGCGGGCAGAACCGAGGCTTCGCCTACGCAAAGTACGGC
D.rerio                 TTTCGCCTCATGATGAACTTCAGCGGGCAGACCCGGGGCTTCGCCTATGCTAAGTACGGT
G.rarus                 TTTCGTCTCATGATGAATTTCAGCGGACAGAACCGAGGCTTTGCCTATGCAAAGTACGGC
M.anguillicaudatus      TTTCGTCTCATGATGAACTTCAGTGGGCAGAACCGCGGCTTCGCCTACGCTAAGTACGGT
O.latipes               TTCCGGCTCATGATGAACTTCAGCGGTCAGAACCGGGGCTTCGCTTACGCCAAATACGGC
P.olivaceus             TTTAGGCTCATGATGAACTTCAGCGGGCAGAACCGTGGCTTTGCATATGCTAAATACAGC
O.mossambicus           TTCCGGCTGATGATGAACTTCAGTGGGCAGAACCGCGGCTTTGCGTATGCCAAATATGGC
                        **  * ** ******** ***** ** **** *** ***** ** ** ** ** **

O.mykiss                AGCCCTGCCTCGGCCGCTGCCGCCATCCGCTCGTTGCATGGCCGTGCCCTGGAGTCAGGG
S.salar                 AGCCCTGCCTCGGCCGCTGCCGCCATCCGCTCGTTGCATGGCCGTGCCCTCGAGTCAGGG
C.gibelio               GACCCCCTCACCGCCTCCACCGCCGTCTTGACCCTGCATCACTACCGTCTGCCGGAGGGG
D.rerio                 GACCCTCTTACAGCCTCGGCTGCCGTCACCACGCTGCATCAGTACCGGCTGCCGGAGGGG
G.rarus                 GACCCCTTCACCGCATCCACCGCCGTCATGACCCTCCATCAGTATCCTCTGCAGGAGGGG
M.anguillicaudatus      GACCAGGTCACGGCCATAACTGCCGTTAAGACCCTCAATCAGCACCGGATTGCGGATGGG
O.latipes               ACGGCCGCCATCGCCAATGATGCCATCCACCTCCTTCACGGCTACCCGCTGGGGCCTGGG
P.olivaceus             TCCTCGGGTTTGGCTGCTGAAGCAATCTGCCTGCTCGATGGCTACATGGTGGAGCAAGGC
O.mossambicus           TCAGCTGCTATAGCTGTTGAAGCCATACGACAGCTGCACGGTCACATGGTGGAGCCTGGC
                                    **       **  *        *  *           *   *   **

O.mykiss                GCACGCCTCAGTGTACGGCGCAGCACAGAGAAACGTCAGCTCTGTCTTGGGGAG------
S.salar                 GCACGCCTCGGTGTACGGCGCAGCACGGAGAAACGTCAGCTCTGTCTTGGGGAG------
C.gibelio               GGCTGCCTGACAGTGCGCAACAGCACAGAGAAGCGGCAGCTGCGTCTGGGGGAT------
D.rerio                 GGCTGCCTGACCGTGCGCAGGAGCACCGAGAAGCGCCAGCTGCGTTTGGGGGAT------
G.rarus                 GTTTGTCTGACGGTTCGCAGGAGCACGGAGAAGCGCCAGCTGCGCCTGGGGGAT------
M.anguillicaudatus      GTTCGATTGATGGTGCGCAGGAGCATGGAGAAGAGGCAGCTGTGGATTGAGGAC------
O.latipes               GCTCGCCTCAGCGTGCGCTGTAGCATCGAGAAGCGCCAGCTCTGCATCCAGAAC------
P.olivaceus             TCCTGCATCAAGGTCCGCCTGAGTATAGAGAAGAGACACCTCTGCATTGAGGAT------
O.mossambicus           TACCGCATCAGTGTACGGCGGAGCACAGAGAAGCGACACCTTTGTATTGGAGGTATTGGA
                            *  *    ** **    ** *  *****  * ** **  *  *

O.mykiss                ---CTGCCCACCAGCACAAGGAGGGAGCAACTGCTGCAGGTGCTGCTGGACTTCTCTGAG
S.salar                 ---CTGCCCACCAGCACAAGGAGGGAGCAACTGCTGCAGGTGCTGCTGGACTTCTCTGAG
C.gibelio               ---CTTCCGACTAGCATAAATCAGGTGGAGTTGCTGAGGATGCTGCGAATGATGTCTGAA
D.rerio                 ---CTGCCCGTCAGCATGAATGAATCGAAGCTGCTGATGGTGCTCCAGATGCTCTCTGAC
G.rarus                 ---CTTCCGACTAACGTGAATCAGACGGAGATGCTGATGGTGCTCCAAATGATGTCTGAG
M.anguillicaudatus      ---CTGCCACCCGACGTGAGCCAGGATGAACTGCTGATGATGCTTCGACAGATTTCGGAG
O.latipes               ---CTGCCAGCTTCCACCAGGCAGGAGGAGCTGCTGCAGGTGCTGCGTTTGCTGTCTGCG
P.olivaceus             ---CTGCCGGCCACCACCAGGCAAGAGGAGCTGCTGGAGGTTCTGCGTGTGCTGACTGAG
O.mossambicus           GGTCTGCCTGCTTCCACTAGACAAGAAGGCATACTGCAGGTGCTGCGTATGCTGGTAGAG
                           ** **      *   *            * ***  * * ** *      *    *

O.mykiss                GGGGTAGAGGGCGTGTCCCTGAG---AGCAGGGCCTCGGGAACAGGGGCTGTCTGCAGTG
S.salar                 GGGGTAGAGGGCGTGTCCCTGAG---AGCAGGGCCTGGGGAACAGGGGATGTCTGCAGTG
C.gibelio               GGGGTGGAGGAGGTCCTGCTGAAGATGGCCGGGCCCAAAGGGAGAGAGTTTGTGGCCCTA
D.rerio                 GGTGTAGAGGACGTCCTGCTCAAGCCACCGGGGCCCAAAGGGAAAGAGGTTGTGGCTCTA
G.rarus                 GGTGTGGAGAATGTCCTGCTGAAGTTGGCCGGGCCAAAAGGGAGAGAGGTCGTGGCTCTA
M.anguillicaudatus      GGCGTGGAGGATGTCACGCTGAAGTTTAGCGGGCCTAAAGGGGGAGATGTCGCGGCTGTA
O.latipes               GGTGTGGAGAGTGTCGCCTTGAA---GGCTGGCCCTGGCATAGAGGGGGTGTCAGCCGTT
P.olivaceus             GGCGTGCAGAAAGTGTCTCTGAC---AGCCGGGCCTGGTATCGAGGGGGTGTCGGCCATA
O.mossambicus           GGGGTGGAGAGAGTTTCCCTGAA---GGCCGGACCTGGTATAGAGGGGGTATCTGCTACT
                        ** **  **   **     * *        ** **          *   *    **

O.mykiss                GTGGTCTATGCCTCCCACCATGCAGCTTCCATGGCCAAGAAGGTGCTGATTGAAGCCTTT
S.salar                 GTGGTCTATGCCTCCCACCATGCAGCTTCCATGGCCAAGAAGGTGCTGATTGAAGCCTTT
C.gibelio               GTGAACTACTCCTCCCACTACGCCGCATCCATGGCCAAGAAAGTGCTTGTGCAAGCTTTT
D.rerio                 GTCAACTACACGTCCCATTACGCCGCATCCATGGCTAAGAAAGTGCTCGTGGAAGCTTTT
G.rarus                 GTGAACTACTCCTCCCACTACGCGGCGTCCATGGCCAAGAAAGTGCTTGTGGAAGCTTTT
M.anguillicaudatus      GTCAGCTACTTCACGCATATCGCTGCATCTATGGCTAACAAAGTATTGGTCGAAGCATTC
O.latipes               GTTGCTTTCTCTTCTCATCATGCAGCCTCCATGGCCAAGAAGGCTCTGGGAGAAGAGTTC
P.olivaceus             GTAGCCTTCTTGTCCCACTACACTGCTTCTATGGCCAAGAAGATGCTGGTTGAAGTGTTC
O.mossambicus           GTTGCTTTCTCATCTCACCATGCAGCTTCTATGGCTAAGAAAGTGCTGGTGGAAGCATTT
                        **    *      * **     * ** ** ***** ** **     *     ***  **
```

```
O.mykiss                AAGAAACGCTTTGGGCTGGCCATCACTTTGAAGTGGCAGTCCTCTTCT---AGGCCCAAG
S.salar                 AAAAAACGCTTCGGGCTGGCCATCACTTTGAAGTGGCAGTCCTCTTCT---AGGCCCAAG
C.gibelio               AGGAAGCAGTACGGCATTTCCATTACTGTGAAATGGATGTCCTTCTCC---AAGTCCAAG
D.rerio                 CGGAACCGGTACGGCATTTCCATCACCGTCAGATGGACTTCCTTCTCC---AAGTCCAAG
G.rarus                 AAGAAGCAGTGCGGTATTTCCATTACTGTCAGTTGGACGTCCTTCTCC---AAGTCCAAG
M.anguillicaudatus      AAGAAGCAGTACGGCATGTCCGTCACTGTAAGATGGATGACTTTCACTGCAAAGTCCAAG
O.latipes               AAGAAGCAGTTCTGCTTAGACATCTCTATCAAGTGGCTCTCTGCAGAG---AAGCCCAAC
P.olivaceus             AAGAAGCAGTTTGCACTGAATATCTCAATCGTATGGCATCCAGACGAA---TCGCTGTCT
O.mossambicus           AAGAAGCAGTTTACAATGTGTGTGTCAGTCAAGTGGCAGCCAACAGAG---AAGCCAAAC
                           ** *  *      *     *  *  *    ***    *            *

O.mykiss                CACGAAGAGCCTCCCAGACCCTCCAA--AACGCCTCCTTC------TCCCAAACCTCCCC
S.salar                 CACGAAGAGCCTCCCAGACCCTCCAA--AACCCCTCCTTCCTCTCCTCCCAAACCTCCTC
C.gibelio               CATGTCGACGAGACTG---GAGAAGATGACCGCTTTACTCCACCTGTTCTGAATCCCCTC
D.rerio                 CGCGTCGAGGACACTCCCCAGGAAGACAGCTGCGTAACCCCACTTGTTCTGAAGCCGCTT
G.rarus                 CGTGTCAAGGAGATGGGACAAGAAGATGACTTCTTTACCACACCTGGTCTAAAACCCCTC
M.anguillicaudatus      CATGTCGAGAAAACGAAACAAGACAACGGCCCCCCTGCCCCACCCAGTCTTAAACCCCTC
O.latipes               CCGGACAAGCCCCC-------TCCTCAGAGAGCTCCTAAAGGCCTGCTGCCGTCCCCCCT
P.olivaceus             CCAAACAAGCCTCCGA-AGAGCCCTCTGGCATCTCCCCTGAAGCCACCATGCCACATCCT
O.mossambicus           CCTGACGAGCCACG----ATGCCCTCAGAAACGTGCAAAGAGCCTGTTGCCGTCACACCT
                        *      *                                                 *

O.mykiss                ACTGCTCCCTC----------------CTCGACAGCCC-CCGGCCTCCCCTGCGCCTCGC
S.salar                 GCTGCTCCCTC----------------CTGGACAGCCC-CCGGCCTCCCCTGCACCTCGC
C.gibelio               CCCAAACCCTC--------CATCACCCCTCCCCACCTG--CACCCTCAGC--CCTTGCTT
D.rerio                 TCTAAACCATCACTCCTG-CATTATGACGTCCCAGCTCACCAGTCTCTGCTTCCTCTCTT
G.rarus                 ACTAAACCGTC--------CCTCTCCCCTCCATGCCTG--TATCCTCAGC--TCTCTCAT
M.anguillicaudatus      TCCAAACCCTT-----------------TTCCCATCTG--CACACTCAGC--CATCCCAT
O.latipes               GAAGCACCTAGGCCA-----------AACCTCCCCTCGGCTGCCCCCACGATTGGCTTCT
P.olivaceus             GAAGTCTCCAC---------------AGCCCTCAGTC--CCGCCTCCACATCTCGCCCAT
O.mossambicus           AGGGCCCCTGCACCACAGTTCTCCACAACCCTCAGGC--CCGCCTTCATTCCTGACCCTC
                               *                                      *

O.mykiss                CCAGC--------------------------------------GTCAGCTCCCTGCCTTC
S.salar                 CCAGC--------------------------------------GTCAGCTCCCTGCCTTC
C.gibelio               C------------------------------------------ATGATGTCCCACCCCAC
D.rerio                 CCGGGCTGTTGGGGGTCCGACCACCAGTGAGCAGAGAGATGAGATGATTCCTCAACCCAC
G.rarus                 C------------------------------------------ATGATGTCCCGGCACAC
M.anguillicaudatus      C------------------------------------------ACATCATTCCGACCCAC
O.latipes               CCCGCA--------------------------------------GTCCCCACAGCCTTC
P.olivaceus             CATTCAC------------------------------------CCACCCCCCCAAGTTTC
O.mossambicus           CCTGCAT------------------------------------CCATACCCGCAGGTTTC
                        *                                                   *      *

O.mykiss                TCCC-------GGGCTGTGAGGGCACCCCCTCCC---------------------ATGGT
S.salar                 TCCC-------GGGCTGTGAGGGCGCCCTCTCCC---------------------ATGGT
C.gibelio               CC-----------ACTAATCCAGCCCTTCTTCCG-----------------------GGC
D.rerio                 CATAATGTCAAGAAATGAGCTGATTCCTCAATCGTCCATAAGGCAGAGAGATGAGATGGT
G.rarus                 CC-----------CACTCTCCAGCCCTTCTTCCG-----------------------GGC
M.anguillicaudatus      AC-----------CACCCCCCAGCCCTTCACCCG-----------------------AGC
O.latipes               TGTAA-----AGCTGTGGGTGGGCC------CCCCACACAG----------CATGATACT
P.olivaceus             TGCAG-----AGCAGTGGGCGGACCTACGGCCTTTTCACAC----------CCTCACTGT
O.mossambicus           TGCAG-----AGCAGTGGGAGGGCCCACTGCTCCTCAGCTC----------TCTCACCCT

O.mykiss                GCACGCTGCTCCTGAATCCCTC---AGGGAGGTGACCGTG------GTGCC---------
S.salar                 GCACGCTGCTCCTGAATCCCCC---AGGGGGGCGACCATG------GTGCC---------
C.gibelio               TGCTGGAGGTCCGGCTGGCCTG---AGAGATGAGACGATG--CCTCGAGCC----CCTGT
D.rerio                 TCCTCAGCTCCCCATAAGGCCG---AGAGATGGGATGGCT--CCCCAATCC----CCCAT
G.rarus                 AGTTGGGGGTCCAACCGCCCCGCCAAGAGATGAGATGATGGCTCCTAACCCTAACCCTGT
M.anguillicaudatus      GGTCGGTGCTCCCGTCACCCAGCCGAGGAACGAGCCGATA------AGTCA---------
O.latipes               CATGTTAAAGGCACCTCTCCTCCCCCCAGGGGACAAGTCA-------TGTT---------
P.olivaceus             CCCCACCACTTCACTTTTTCCTCTTCGCAGGGGGACCGAG-------TGCC---------
O.mossambicus           ACATGCTCTTTTCCCGATTCCTCCACCCAAGGCCATCTTG-------TATT---------
                                           *           *
```

```
O.mykiss                TCCTGTGGATGCAGCAGCCCTGCTCCAGGGTGTGTGTGAGGTGTACGGGCAGGGGAAGCC
S.salar                 TCCTGTGGATGCAGCAGCCCTGCTCCAGGGTGTGTGTGAGGTGTACGGGCAGGGGAAGCC
C.gibelio               GAATCAGGATGCCGTGTCTCAGCTGCAGTGGATATGTGAGCTGCACAGACTCGGCACTCC
D.rerio                 TAGTCTCGACGCCGTGTCTCATCTGCAGTGGATGTGCGAGGTCAACAGACTCGGCTCTCC
G.rarus                 GAACCGTGACACCGTATCTCAGCTGCAGAGTATGTGCGAGGCAAACAGACTCGGTGCGCC
M.anguillicaudatus      AAGCCGTGACGCTGCGTCTCAGCTGAAGTGGCTCTGCGAGGTGAACAAACTCGGAACGCC
O.latipes               TTCTGTGTCCCCGGTGCTGTTGCTGCGGAAGCTGAGTGAGGCGAGTGGGTGGGGGATCC
P.olivaceus             TGTTGAATCCCCAGTGATGCTCCTGAGTAAGGTGTGTGAGGTGACGGGGGTTGGTCAGCC
O.mossambicus           TGCAGCATCCCCAGTGATGTTTCTCAGTAAGCTGTGTGAGACAGCAGGTGTTGGCCAGCC
                                   * *        **        *  * ***            **    **

O.mykiss                CCTCTATGACCTGCAGTACCGCCACATGGGGCCTGACGGG--TTCCTGTGCTT-CAGCTA
S.salar                 CCTCTATGACCTGCAGTACCGCCACATGGGGCCTGACGGG--TTCCTGTGCTT-CAGCTA
C.gibelio               GCAGTATGAAGTCCGTTTCCATCGCACCAGCTCCGACGGC--TTCCTCTACTT-CAACTT
D.rerio                 GCAATATGAAGTCCACTTCCATCACGCGGCTCCTGACGGA--TTCCTCTACTT-CGCCTT
G.rarus                 ACAGTACGAAGTCCGTTACCATCACACTGGCCCAGACGGGCTTTCTTCTACTTTCGCTTT
M.anguillicaudatus      ACGATACGAAGCACGATACAATCACATCGGTCCCGACGGC--TTTCTCTATTT-CACGTA
O.latipes               ACACTATGAGATGCTTTTTAGCCACGCCGGCCCAGATGGA--TTTCTGTACTT-CACCTA
P.olivaceus             ACGCTATGAGATGTCCTACAGCCACACTGGAAGAAATGGA--TTCCTCTACTT-CGACTA
O.mossambicus           GTTTTATGAAGTGCAGTACAGCCACGCTGGGCCTGATGGA--TTCCTCTACTT-CTCCTA
                            ** **       *     * *          * **   **  * *  ** *   *

O.mykiss                CCGGGTGTATGTGCCGGGGCTGGCCACACCCTTCACTGGGATGGTGCAGACACTGCCCGG
S.salar                 CCGGGTGTATGTGCCGGGGCTGGCCACACCCTTCACTGGGATGGTGCAGACTCTGCCCGG
C.gibelio               CAAAGTCCTGGTCCCAGGCCTTCTTTTTCCTCTGAACGGGGTTATCCAGATCCTTCCAGG
D.rerio                 CAAAGTGCTGATCCCAGGCCTGCCGCTGCCCCTGTATGGGTTCGTCCAGATCCTGCCAGG
G.rarus                 CAAAGTGCTGATCCCAGGCCTCCTTCTGCCTCTGTTCGGGGCTGTCCAGATCCTTCCAGG
M.anguillicaudatus      CAAGGTCATGATCCCAGCGCTTTCTTTTCCCCTGTACGGTTCTGTCCAGATCCTGCCAGG
O.latipes               CAAGGTGCACGTCCCCGGAGCCCCCACCACCTTCAGGGGCTTCGTCATGATTTTACCAGG
P.olivaceus             CACAGTGTGTATCCCCAGGATAACCACTCCTTTCAAAGGGCTGGTCATGACCTTGCCAGG
O.mossambicus           TAAGGTGCAGATCCGCGGGATCACTGCCGCTTTCAAGGGGTTGGTCATGATCTTACCGGG
                            **     * *               *  *    **     *   **   * ** **

O.mykiss                CCCCACCCCTGGAGCCATACAGGAAGAGGCTCGCAGAGCTACAGCCCAGCAGGTCCTCAG
S.salar                 CCCCACCCCTGGAGCCATACAGGAAGAGGCTCGCAGAGCTACAGCCCAGCAGGTCCTCAG
C.gibelio               CACCAGCATTCAAGCCATGAAGGCAGAAGTTTATCGAGCCGCTGCCGAGCAGGTGATCCA
D.rerio                 CACCAGCGCACGAGCCATGAAGAGTGAAGTTTACCGGGCCGCCGCTGAGCAGGTGATCCA
G.rarus                 CACCAGCGCTCAAGCCATGAAGGGGGAAGTTTGTCGAGCCGCCGCCGAGCAGGTGATCCA
M.anguillicaudatus      TACGAGCGCTCGGGCCATGAAGGGGGAAGTTCATCGAGCCACTGCTGAGCAAGTGATCCA
O.latipes               ACACTGCACGTCCACCATGCTGGAGGAGGCCAGGAGGGCTGCTGCCCAGCAGGTCCTGCA
P.olivaceus             ACCCACTGCCAGCACCGTGCAGGAGGAAGCTGGGCAGGCTGCAGCCCAACAGGTCCTGAA
O.mossambicus           TCCTAATGTCAGCACCATGCTAGAGGAGGCTCAGAAGGCTGTAGCCCAGCAGGTCCTGCA
                                      ** *       ** *        **     **  * ** **  *

O.mykiss                CGCTCTGTACAGGG-CCTGA----------------------------------------
S.salar                 CGCTCTGTACAGGG-CCTGA----------------------------------------
C.gibelio               AACCATGTGTAAAG-TCTCAAACTTGCGACCTTTCTAA----------------------
D.rerio                 AACCTTGTGCCGAG-TCTCAAATTTACGGCCTTTCTAA----------------------
G.rarus                 AACCATGTGCAAAGGTCTCAAATTTGCGACCTTTCTAAAATTTGAGCTGTTTTTGCTAAT
M.anguillicaudatus      AACCATATACACGG-CTGCGCGTTGGCAACCTTTCTAA----------------------
O.latipes               GAAGTTGTGCAGCA---GCGGTTTGAGCGCCTGA---------------------------
P.olivaceus             GAGAATTTACAACA---ACCAGTGA-----------------------------------
O.mossambicus           GAAGATGTACAACA---CTGGTCTCACACACTAA---------------------------
                             * *

O.mykiss                ----
S.salar                 ----
C.gibelio               ----
D.rerio                 ----
G.rarus                 GTAA
M.anguillicaudatus      ----
O.latipes               ----
P.olivaceus             ----
O.mossambicus           ----
```
